# Modeling and Fundamental Dynamics of Vacuum, Gas, and Antisolvent Quenching for Scalable Perovskite Processes

**DOI:** 10.1002/advs.202308901

**Published:** 2024-02-02

**Authors:** Simon Ternes, Felix Laufer, Ulrich W. Paetzold

**Affiliations:** ^1^ CHOSE–Center for Hybrid and Organic Solar Energy Department of Electrical Engineering University of Rome “Tor Vergata” via del Politecnico 1 Rome 00133 Italy; ^2^ Light Technology Institute (LTI) Karlsruhe Institute of Technology (KIT) Engesserstrasse 13 76131 Karlsruhe Germany; ^3^ Institute of Microstructure Technology (IMT) Karlsruhe Institute of Technology (KIT) Hermann‐von‐Helmholtz‐Platz 1 76344 Eggenstein‐Leopoldshafen Germany

**Keywords:** antisolvent quenching, crystallization gas quenching, perovskite, photovoltaics, supersaturation, vacuum quenching

## Abstract

Hybrid perovskite photovoltaics (PVs) promise cost‐effective fabrication with large‐scale solution‐based manufacturing processes as well as high power conversion efficiencies. Almost all of today's high‐performance solution‐processed perovskite absorber films rely on so‐called quenching techniques that rapidly increase supersaturation to induce a prompt crystallization. However, to date, there are no metrics for comparing results obtained with different quenching methods. In response, the first quantitative modeling framework for gas quenching, anti‐solvent quenching, and vacuum quenching is developed herein. Based on dynamic thickness measurements in a vacuum chamber, previous works on drying dynamics, and commonly known material properties, a detailed analysis of mass transfer dynamics is performed for each quenching technique. The derived models are delivered along with an open‐source software framework that is modular and extensible. Thereby, a deep understanding of the impact of each process parameter on mass transfer dynamics is provided. Moreover, the supersaturation rate at critical concentration is proposed as a decisive benchmark of quenching effectiveness, yielding ≈ 10^−3^ − 10^−1^s^−1^ for vacuum quenching, ≈ 10^−5^ − 10^−3^s^−1^ for static gas quenching, ≈ 10^−2^ − 10^0^s^−1^ for dynamic gas quenching and ≈ 10^2^s^−1^ for antisolvent quenching. This benchmark fosters transferability and scalability of hybrid perovskite fabrication, transforming the “art of device making” to well‐defined process engineering.

## Graphical abstract

Perovskite photovoltaics are very popular due to their excellent properties for commercial tandem photovoltaic applications. When printing these photovoltaics, quenching processes are pivotal for fabricating high‐quality absorber layers. This work presents the first open‐source software for quantitative modeling of mass transfer dynamics and determination of supersaturation rates in vacuum, gas, and antisolvent quenching–enabling a reproducible and scalable perovskite fabrication.

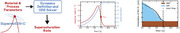

## 1 Introduction

Hybrid perovskite photovoltaics (PVs) have attracted great research interest within the last 15 years–given their high potential for future renewable energy production.^[^ ^1^, ^2^, ^3^ ^]^ The reason is not only that power conversion efficiencies (PCEs) of perovskite solar cells (PSCs) skyrocketed from 3.2% in 2009 to over 25% to date; also the solution processability of the hybrid perovskite material class is considered a decisive factor for their imminent market entry, promising very cost‐effective fabrication.^[^ ^4^ ^]^ Possibly the most advantageous property of perovskites is the fact that their bandgap can be tuned over a wide range via the chemical composition,^[^ ^5^ ^]^ making them an ideal candidate for tandem PV applications with other technologies such as silicon,^[^ ^6^ ^]^ CIGS^[^ ^7^ ^]^ or even perovskite^[^ ^8^ ^]^ itself. These tandem modules are very likely to become the first mass‐produced commercial PV technology to overcome the Shockley‐Queisser limit in PCE.^[^ ^6^, ^9^ ^]^

However, there is still a fundamental hurdle for perovskite PVs to reach industrial reliability as it is known among researchers for its insufficient reproducibility.^[^ ^10^, ^11^, ^12^ ^]^ This is because perovskite crystallization is very sensitive to environmental parameters.^[^ ^11^, ^13^ ^]^ Even a slight deviation in process parameters can have severe impacts on the morphology of polycrystalline perovskite thin‐films used as absorbers in PSCs. These difficulties affect not only the reproducibility of fabrication routines but also their scalability. If reproducing defect‐less thin‐films on a small sample area is challenging, the likelihood of success decreases even further in larger areas.^[^ ^14^, ^15^, ^16^, ^17^ ^]^ Therefore, it is not surprising that, as compared to concurrent technologies, performance losses in PSCs with increasing device area are still substantial.^[^ ^12^ ^]^

To understand the emergence of the above‐described challenge, a close look at the solution‐based processing of perovskite thin films, and in particular perovskite crystallization, is pivotal. If the perovskite solution dries in an uncontrolled way, a rich crystalline microstructure forms featuring ribbons,^[^ ^18^ ^]^ fractional crystallites,^[^ ^19^ ^]^ and porous, circular domains^[^ ^20^ ^]^ – impeding a full coverage of the substrate. All pinholes in the absorber layer will, in turn, drastically decrease the achievable PCE.^[^ ^21^ ^]^ Commonly, perovskite nucleation, thin film formation, and crystal growth are controlled by so‐called quenching methods.^[^ ^12^ ^]^ The basic principle of quenching is the rapid increase in supersaturation of the perovskite solution right at the onset of crystallization. By this means, a high nucleation rate is achieved, such that the resulting film morphology covers the entire surface area with a densely packed, homogeneous thin‐film of nuclei–a precondition for achieving high PSC performances.^[^ ^18^, ^22^ ^]^

We categorize three common quenching methods in state‐of‐the‐art PSC fabrication: a) gas quenching,^[^ ^17^, ^23^, ^24^ ^]^ that is forced convection of a drying gas over the solution film b) vacuum quenching,^[^ ^8^, ^15^, ^16^, ^25^, ^26^ ^]^ where the substrate is placed in a vacuum chamber, whose atmosphere is rapidly pumped out c) antisolvent quenching,^[^ ^27^, ^28^, ^29^, ^30^ ^]^ that is immersion of the substrate with another solvent or bathing in the solvent (it is noted that in some cases multiple of these quenching methods are combined^[^ ^31^ ^]^). From its first occurrence in literature,^[^ ^27^ ^]^ quenching has not only been the dominant processing step for most record solution‐processed PSCs, but it has also enabled the unprecedented rise in PCE of certified champion devices mentioned above.^[^ ^32^ ^]^ When comparing the processing of perovskite solar cells with concurrent solution‐processable PVs,^[^ ^33^, ^34^ ^]^ the quenching process marks the crucial difference. Considering this fact, it is astonishing that there are very few reports on the differences induced by the specific quenching method in large‐scale solution printing setups.^[^ ^35^ ^]^ Further, different quenching methods are only compared on a phenomenological basis by investigating readily crystallization perovskite films and PCEs of fabricated PSCs. Frameworks for predicting the morphology evolution of perovskite thin films as a function of drying rate or supersaturation were presented by Ronsin et al.^[^ ^36^, ^37^ ^]^ and Michels et al..^[^ ^38^ ^]^ However, an application‐oriented in‐depth quantitative analysis of the mass transfer dynamics for predicting the apparent supersaturation or drying rate during quenching is missing.

Since the fundamentals of mass transfer dynamics during quenching are unknown, it is no wonder that the reproducibility of reported processes proves insufficient. For transferring process routines to a new laboratory, coating or drying machine, researchers must re‐optimize the process within a larger parameter space to achieve comparable results. A testable and reproducible quantitative description of experiments is urgently needed. In response, leveraging extensive knowledge from the field of chemical engineering,^[^ ^39^, ^40^ ^]^ we present the first quantitative modeling framework for describing mass transfer dynamics in all common quenching methods used in perovskite photovoltaics–revealing an astonishingly high degree of commonality and simplicity of the involved mass transfer dynamics. Evidence for the models' prediction power was provided by interferometric measurements of film thickness decrease on evaporation earlier.^[^ ^20^, ^22^, ^41^ ^]^ Herein, the testing of models is extended to vacuum quenching for the first time. In particular, this work delivers three main novelties to the perovskite research community: First, we provide an open‐source software framework for calculating quantitative mass transfer dynamics–expanding our previously developed models to the other two quenching methods, namely vacuum quenching and antisolvent quenching. This way, we deliver the capability to understand similarities and differences between the fundamental dynamics of these quenching methods, which is unprecedented in current literature. Second, the application of our models enables researchers and technologists to understand the exact impact of every process and material parameter, giving them the ability to forecast the outcome of their experiments and specific fabrication tools. Third, leveraging the results of this work, researchers can determine and compare the supersaturation rates achieved within their fabrication setups. Due to their high decisiveness for perovskite morphology evolution, supersaturation rates are an ideal benchmark of quenching effectiveness, ensuring standardization and comparability of chemical processing. We expect the three results detailed above to yield significant advances in scalability and reproducibility of perovskite coating and drying initiating a journey from the “art of device making” to well‐defined process engineering.

## 2 Results and Discussion

### 2.1 Introduction of Modeling Framework

**Figure 1:**
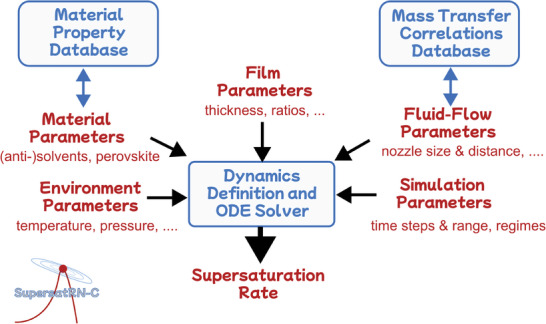
Schematic of SupersatRN‐C open source software. The software modules are shown in blue and the parameters/settings in red. The logo of the software is depicted in the bottom left corner.

In the following, we introduce our modeling framework for the most common quenching methods (see **Figure** **1**). The framework is released as an open‐source software package, “SupersatRN‐C”, that is modular and extensible. SupersatRN‐C comprises two main database modules that feed structured information about the materials and mass transfer correlations into the main module, which calculates the solutions to the respective dynamic equations. In the following sections, we present the explanations of these key dynamic equations and discuss in detail the assumptions made as well as the boundaries of validity. Thereby, the limitations of the model are transparent, and future generations of scientists can build on this framework by extending or detailing certain aspects. This idea of further development is explicitly encouraged by the full disclosure of the methodology and codes presented in this work.

#### 2.1.1 Quantification of Quenching Effectiveness by Supersaturation Rates

When describing quenching, hybrid perovskite researchers often motivate LaMer's model of particle formation introduced in 1950.^[^ ^35^, ^42^, ^43^, ^44^, ^45^, ^46^, ^47^ ^]^ The history of citing LaMer's model for explaining dynamic crystal nucleation and growth, and, in particular, “burst nucleation”, has recently been questioned by Whitehead et al..^[^ ^48^, ^49^ ^]^ However, irrespective of the question, of whether LaMer's model predicts correctly the nucleation and growth dynamics in perovskite precursor solutions, there is an overwhelming consensus within the community: Supersaturation in the perovskite solution film must be increased promptly during perovskite thin film formation to enable a densely‐packed film morphology of small crystalline nuclei. In other words, all quenching processes share a common purpose: Reaching a very high supersaturation rate right at the moment when the critical concentration for perovskite nucleation is reached. Experimental evidence of this statement to be effective in predicting perovskite morphology formation was already provided by our previous works as well as those of other groups,^[^ ^20^, ^22^, ^50^, ^51^, ^52^ ^]^ which is why we consider it a reasonable assumption herein. According to textbooks,^[^ ^53^ ^]^ one possible definition of the supersaturation is

(1) $$\sigma =\ln\left(C/C_{0}\right)$$

where *C* [mol m^−3^] is the molar solute concentration and *C*_0_ [mol m^−3^] is the equilibrium molar concentration of solute (the concentration at which the material neither precipitates nor dissolves with time). With this definition, the above‐described goal of quenching can be defined as maximizing the temporal derivative at the time when the critical supersaturation/concentration is reached (indicated by the subscript “crit.”),

(2) $${\dot{\sigma }}_{\mathrm{crit}.}={\left.\frac{\dot{C}}{C}\right|}_{\mathrm{crit}.}-{\left.\frac{\dot{C_{0}}}{C_{0}}\right|}_{\mathrm{crit}.\;}$$

This relation highlights that the supersaturation rate can be increased either by increasing the solute concentration, *C*, or by decreasing the equilibrium concentration, *C*_0_, over time. In fact, the different quenching concepts make use of either of these routes: Vacuum quenching and gas quenching increase *C* by evaporation of solvent and *C*_0_ is assumed constant, while antisolvent quenching predominantly reduces *C*_0_ by diffusion of antisolvent and *C* is assumed constant (details of the validity of these assumptions follow in Sections 2.1.3. and 2.1.4.). We note that, for effective quenching, $\dot{\sigma }$ must be as high as possible at the time of the nucleation onset (we approximate the nucleation as instantaneous), but it is rather irrelevant before and after that time.^[^ ^20^ ^]^ We further note that the critical concentration, *C*_crit._, is commonly greater *C*_0_. The reason is the energy barrier associated with nucleus formation.^[^ ^53^ ^]^

#### 2.1.2 Determination of Solute and Equilibrium Concentrations

In order to calculate supersaturation rates, we need to determine the solute and equilibrium (solute) concentrations. We consider a film of dissolved perovskite precursor materials in a solution with *n* different solvents and assume that all of its components are, at all times, homogeneously mixed (there are no concentration gradients). We designate the number of molecules of solvent *k* in the film as *N_k_* (*t*) [mol] and the number of perovskite unit cells as *N*_pvk_ [mol]. Considering these assumptions, the total thickness of the film is given by

(3) $$d\left(t\right)=d_{\mathrm{pvk}}+\sum_{k=1}^{n}d_{k}\left(t\right)=\frac{N_{\mathrm{pvk}}}{\delta A\;{\tilde{\rho }}_{\mathrm{pvk}}}+\sum_{k=1}^{n}\frac{N_{k}\left(t\right)}{\delta A{\tilde{\rho }}_{k,l}}$$

where *d*_pvk_ [m] is the part of the total film thickness which is attributed to the perovskite unit cells, *d*_k_[m] is the part of the total film thickness occupied by solvent *k*, δ*A* [m^2^] is a small area element, ${\tilde{\rho }}_{\mathrm{pvk}}\left[{\mathrm{mol}\;m}^{-3}\right]$ is the molar density of the perovskite unit cells (approximated as constant) and ${\tilde{\rho }}_{k,l}\left[{\mathrm{mol}\;m}^{-3}\right]$ is the molar density of the liquid solvent *k*. By employing *d*(*t*) from Equation (3), we calculate the solute concentration,

(4) $$C\left(t\right)=\frac{N_{\mathrm{pvk}}}{d\left(t\right)\;\delta A}$$

For gas and vacuum quenching, we assume a constant equilibrium concentration *C*_0_ (and thus ${\dot{C}}_{0}=\;0)$ because, despite the removal of the solvent, the solution is unaltered. We estimate *C*_0_ by weighing a certain amount of precursor chemicals and adding small amounts of solvent until the solution appears transparent, indicating that enough solvent is present to dissolve visible material clusters (see Note S1 and Figure S1, Supporting Information). For antisolvent quenching, we repeat the same experiment, using a mixture of antisolvent and solvent with a well‐defined volume ratio (see Figure S2, Supporting Information). This is done for varying volume ratios to extract a correlation between volume ratio and equilibrium concentration. As a result, in Figure S2 (Supporting Information), we demonstrate that the addition of antisolvent lowers the equilibrium concentration approximately as a linear function of the antisolvent‐to‐solvent volume ratio, that is according to

(5) $$C_{0}\left(t\right)={\bar{C}}_{0}-q_{C}\frac{N_{j}\left(t\right)}{\sum_{k\neq j}s_{\mathrm{jk}}N_{k}\left(t\right)}$$

where ${\bar{C}}_{0}\left[\mathrm{mol}\;m^{-3}\right]$ is the equilibrium concentration of the pristine solution, *q*_C_ is the equilibrium solute concentration decline [mol m^−3^] and $s_{\mathrm{jk}}={\tilde{\rho }}_{j,l}/{\tilde{\rho }}_{k,l}$. The summation is carried out over all solvents except the used antisolvent *j* (this empirical relation is, evidently, only valid until *C*_0_(*t*) = 0, which is however not relevant for future calculations, since it would imply infinite supersaturation, and crystallization is assumed to occur much earlier, that is when critical supersaturation is reached).

We note that we expect the critical concentration to decay similarly to Equation (5) when antisolvent is added, since the nucleation will be strongly promoted by the presence of the antisolvent. In the following, we assume that the critical concentration declines with the same *q*_C_ as the equilibrium concentration according to Equation (5), however with a constant offset starting at the critical concentration of the pristine solution ${\bar{C}}_{\mathrm{crit}.}$ (see right axis on Figure S2, Supporting Information) which we estimate from interferometric drying measurements (see Figure S3, Supporting Information). This is the simplest possible assumption–requiring no additional model parameters, and thus avoiding over‐parametrization. We expect that, in the future, detailed knowledge of perovskite nucleation will replace this working hypothesis.

When antisolvent is added, the nucleation is assumed to start as soon as *C* exceeds *C*_crit._. Consistently, this will occur at a much smaller *C* than in the case where no antisolvent is added. We note that, although potentially of great merit for future investigation, we treat neither microscopic nucleation mechanisms, solute‐solvent complexes nor coordination chemistry herein, and deliberately focus on liquid‐liquid and liquid‐gas mass transfer. We are aware that summarizing the properties of a complex solution solely in the parameters *C*_0_ and *C*_crit._ (and solvent activities γ_i_) is a stark simplification that will restrict the prediction capability of the developed models. However, given limited experimental evidence based on interferometric thickness measurements and commonly known correlations of mass transfer, simplification is necessary to avoid over‐parameterization. In the future, advanced models of nucleation and crystal growth could be included in the models tested with more advanced characterization techniques.

#### 2.1.3 Dynamic Equation of Liquid–Gas Phase Mass Transfer for Vacuum Quenching and Gas Quenching

**Figure 2:**
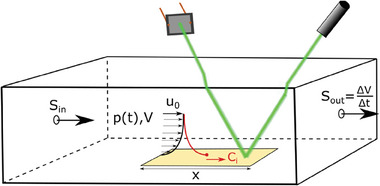
Vacuum‐chamber of volume *V* with a sample of length *x* coated with perovskite solution in its center as typically used in state‐of‐the‐art vacuum quenching^[^ ^8^, ^15^, ^26^, ^63^, ^64^, ^65^ ^]^. The pumping of air out of one side of the chamber with the rate *S*_out_ = Δ*V* / Δ*t* induces a laminar air flow over the sample and a (concentration) boundary layer forms. Optionally, a leak can be added on the opposite side, enabling an inflow of air with rate *S*_in_. In order to test the dynamic equations, we added the ability of interferometric thickness measurements with a green laser and a photodiode (for further details see Note S4, Supporting Information).

First, we elaborate on how the mass transfer dynamics over a liquid‐gas interface can be described. As shown in earlier works,^[^ ^20^, ^22^, ^41^ ^]^ the flux of gaseous solvent away from the film surface can be written with the linear approach of drying as

(6) $${\dot{N}}_{i}\left(t\right)=-\delta A\beta_{\mathrm{ig}}\left(t\right)\frac{\gamma_{i}p_{i}^{*}}{RT}\frac{N_{i}\left(t\right)}{\sum N_{k}\left(t\right)+N_{\mathrm{pvk}}}\;\mathrm{for}\;i=1,\;\cdots \;n$$

where β_ig_(*t*) [m s^−1^] is the so‐called mass transfer coefficient (see Note S2, Supporting Information), $p_{i}^{*}$ [Pa] is the vapor pressure of the pure solvent *i*, γ_i_ is the activity of solvent *i* in the mixture (ratio between the vapor pressure of pure solvent and vapor pressure in the mixture), *R* = 8.31 J K^−1^mol^−1^ is the ideal gas constant, *T*[*K*] is the temperature assumed constant everywhere (we test the validity of this “isothermal assumption” in Note S3, Supporting Information) and δ*A* is a small area on which β_ig_(*t*) is assumed spatially homogeneous (or designates a lateral average value). In a nutshell, β_ig_ determines the slope of the concentration gradient of gaseous solvent (see red line above the film in **Figures** **2**,**4**, and 7), which governs the solvent extraction rate. We note that the main underlying assumption for these equations to hold is that the drying process is fully “gas phase controlled”.^[^ ^20^, ^54^ ^]^ That is to say, there is no gradient of solvent concentration present within the film due to the fast diffusion of liquid solvents within the thin film (see justification of this assumption in Ref.[#0041]).

A short note on the spatial homogeneity of β_ig_: In this work, we mainly employ lateral average values of β_ig_ because literature knowledge on Sherwood numbers predominantly focuses on integrated, average values.^[^ ^39^, ^55^ ^]^ An exception is dynamic gas quenching, which is based on one of our earlier works,^[^ ^20^ ^]^ where we have exactly determined local values of β_ig_. The homogeneity of β_ig_ evidently impacts the homogeneity of the reached supersaturation rate and thereby the scalability of the respective quenching techniques (which is consistent with our earlier work).^[^ ^20^ ^]^ In Note S2 (Supporting Information), detailed information on the homogeneity of Sherwood numbers is provided and, whenever possible, the homogeneity of supersaturation rates will be analyzed for each distinct quenching method (Sections 2.2.1–2.2.3).

Another remark on the solvent activity: Herein, we investigate two main solution formulations: i) Methylammonium lead iodide (MAPI) dissolved in N,N‐Dimethylformamide (DMF) due to its extensive use as a reference system^[^ ^56^, ^57^, ^58^, ^59^ ^]^ and its simple composition and ii) a more complex double cation solution with a mixture of DMF, DMSO and GBL inspired by previous works on vacuum quenching^[^ ^15^, ^16^ ^]^ (see Experimental Section, for detailed description of solutions). For all solvents, we assume constant solvent activity, γ_i_ ≤ 1. We have shown before that this is true for the example MAPI where γ_DMF_ = 1, such that the vapor pressure equals the vapor pressure of the pure DMF.^[^ ^22^ ^]^ We have also demonstrated that for a triple cation perovskite solution, we obtain two drying regimes, one initial regime with γ_i_ = 1 for all solvents and a second drying regime with γ_DMSO_ ≈ 0.025 at the end of the drying process, which we attributed to the formation of an intermediate phase.^[^ ^20^ ^]^ We elaborate in Note S4 (Supporting Information) that we face a similar regime‐based drying for the double cation precursor investigated herein (see Experimental Section, for a detailed description of solutions). In particular, we show that, in this solution, γ_DMSO_ = 0.16 and γ_*GBL*_ = 0.11 at the end of drying. We note that, as long as there is no detailed theory of solvent activity in perovskite precursor films available, the software framework exhaustively describes the behavior of perovskite solution drying via these regime‐based solvent activity changes. Introducing more model parameters is not sensible, at this state, as it would lead to overfitting of the data. Since the software framework is extensible, a more detailed theory of solvent activity of a mixture model (like for example Fluory–Huggins^[^ ^60^ ^]^) to describe the reduction of solvent activity could be added in the future. We further note that we elaborate in Section 2.1.6. how new solution formulations can be added easily to the provided simulation framework, making our methodology compatible with a large variety of solutions with different parameters.

#### 2.1.4 Main Dynamic Equation for Liquid–Liquid Phase Mass Transfer in Antisolvent Quenching

Having described in the previous section the dynamic equation of liquid‐gas phase mass transfer, we detail here the liquid‐liquid phase mass transfer for antisolvent quenching. We assume that, due to the initial drying before the start of the quenching, the perovskite solution has a much higher viscosity than the ejected antisolvent. As a consequence, the flow of antisolvent does not deform or displace the perovskite solution film. Interestingly, in contrast to gas quenching, antisolvent quenching comprises the diffusion of two components: a) the diffusion of solvents upwards as in gas quenching and b) the diffusion of antisolvent downwards. Thus, the boundary between the perovskite solution film and the antisolvent blurs successively. This implies that there is no longer any self‐evident meaning of the terms “inside the solution film” and “outside the solution film”. To mitigate this aspect and to re‐establish an analogy to the gas quenching case, we simplify the problem and assume only two volume elements in the diffusion process. The bottom volume element is addressed as “inside the film” (drawn later in Section 2.2.4 as a yellow bar in **Figure** **7**) and the top one “outside the film” (drawn later in Section 2.2.4 as the light blue area in Figure 7). In addition, we neglect the diffusion of the perovskite components into the top volume element, since for many commonly applied solvents, precursor salts are not soluble in the antisolvent and therefore remain close to the polar solvents in the bottom^[^ ^28^ ^]^ (while it is in principle possible to model the diffusion of precursor salts upward, there is no gain in this work for this complexification of models, since it would impact the diffusion of solvents only slightly and crystallization mechanisms are not treated herein). In analogy to gas quenching, we focus on the number of molecules of antisolvent *j* and the number of molecules of solvent *k* “inside the film”, which we designate by *N_j_* and *N_k_*, respectively. This enables the calculation of an analogous “film thickness”, *d*, with Equation (3). We assume a conservation of volume flow during diffusion (that is an invariant density of solvents before and after mixing). Thus, the volume of antisolvent *j* flowing into the film equals the total volume of solvent flowing out of the film, which can be written as

(7) $${\dot{N}}_{j}\;\left(t\right)=-\sum_{k\neq j}s_{\mathrm{jk}}{\dot{N}}_{k}\left(t\right)$$

where $s_{\mathrm{jk}}={\tilde{\rho }}_{j,l}/{\tilde{\rho }}_{k,l}$. This is equivalent to stating that the “film thickness” is constant, implying $\dot{d}=0$ or *d* (*t*) = *d*_0_ (and thus $\dot{C}=0$). Integrating Equation (7) over time, we get

(8) $$N_{j}\;\left(t\right)=\sum_{k\neq j}s_{\mathrm{jk}}\left(N_{k,0}-N_{k}\left(t\right)\right)$$

where *N_k_* (*t* = 0) = *N*_k,0_ and *N*_j_ (*t* = 0) = 0 was assumed as a sensible boundary condition. Successively, we can calculate the rate of solvent molecules diffusing through the “film surface” very similarly to in gas quenching with a linear approach, ${\dot{N}}_{i}=-\delta A\beta_{\mathrm{ij}}\;{\tilde{c}}_{i}$ with ${\tilde{c}}_{i}\left[{\mathrm{mol}\;m}^{-3}\right]$ being the molar concentration of solvent *i*, yielding

(9) $${\dot{N}}_{i}\left(t\right)=-\frac{\beta_{\mathrm{ij}}}{d_{0}}N_{i}\left(t\right),\;\mathrm{with}\;i=1,\text{…},n\;\mathrm{and}\;i\neq j$$

The mass transfer coefficient, β_ij_, is calculated with Sherwood correlations analogously to gas quenching (see Note S2, Supporting Information). By dividing the above equation by $\delta A{\tilde{\rho }}_{i,l}$, we directly find a dynamic equation for the thickness part of the respective solvent *d_i_* (the number of antisolvent molecules and their thickness part of antisolvent is calculated using Equation (8)).

#### 2.1.5 Movement of Substrate

In some perovskite quenching processes, additional complexity is introduced by the relative movement of the substrate during quenching. We distinguish three modes of movement: i) no movement of the substrate (static) ii) linear movement of the substrate and iii) rotational movement of the substrate. Evidently, the rotational case iii) is the most challenging to describe mathematically and difficult to validate because the sheering dynamics of the solution film superpose with the evaporation.^[^ ^61^ ^]^ In this work, we mainly investigate cases (ii) for gas quenching and, additionally, (i) for all other quenching methods. There are two significant differences between (i) and (ii). First, if the mass and heat transfer is inhomogeneous: One element of the film will experience a temporally evolving mass transfer due to the linear movement in case (ii). Second, the relative velocity of the quenching medium in relation to the substrate is slightly modified in case (ii). In this report, we assume that the second effect is neglectable, which is to say that the movement of the quenching medium is much faster than the movement of the substrate. The exact magnitude of this effect can be analyzed in future works. Finally, we would like to give a short note on case (iii): The radial flow of solution stagnates after a certain initial time in the spin coating.^[^ ^62^ ^]^ Hence, by focusing only on this time regime, the here‐presented models can be applied to spin coating, as well, as long as the correct Sherwood correlation is chosen (see Sherwood correlations in Note S2, Supporting Information).

#### 2.1.6 Software Engineering

The modeling framework is implemented as an open‐source library called “Supersaturation Rate Numerical‐Calculator (SupersatRN‐C)” published on GitHub under the GPLv3 public license, delivering one of the main results of this work (the URL and more details are provided in Section 4.3). As schematized in Figure 1, SupersatRN‐C consists of three modules. The material property database (“materials.py”, “material_properties.py”) contains functions and data to calculate the densities, diffusion coefficients, viscosities, etc. of different chemical compounds (all sources are indicated as comments). For adding a new material system, the lists can be simply extended with their material properties from literature. It also includes the experimental data of the equilibrium and critical concentration decline upon the addition of antisolvent according to Equation (5). The mass transfer correlations database (“correlations.py”) solely comprises different, commonly known correlations for calculating the (local) Sherwood number of mass transfer for different flow jets in dependence on the fluid parameters such as the nozzle geometry, distance from the film and velocity of the fluid (see Note S2, Supporting Information). Eventually, the main module (“__main__.py”) defines the differential equation to be solved by the chosen quenching method, given a correlation and the composition of the initial film are specified. It requires all parameters indicated in red in Figure 1 to be correctly set to produce a sensible, quantitative prediction of the supersaturation rate at critical concentration, as will be detailed in the paragraphs below. Remarkably, the object‐oriented structure of the code reveals the astonishingly high degree of commonality between the different quenching methods detailed below (the classes representing different quenching methods are derived from the same parent and only slight changes are required for the implementation of each respective quenching method). The other files provided in the repository are concerned with the testing of the provided functionality and production of the results plots analyzed below.

### 2.2 Application of Modelling Framework to All Common Quenching Methods

In this section, we demonstrate the application of the above‐described software framework, SupersatRN‐C, on a characteristic experiment for each quenching method. Most importantly, we input experimental parameters as used in real‐world perovskite thin film processing (detailed in Experimental Section; **Table** **1**). We start the analysis with vacuum quenching and present a direct test of the model with interferometric measurements, therefore validating the chosen modeling concepts (Similar validations were conducted in earlier works for gas quenching.^[^ ^20^, ^22^, ^41^ ^]^). As a result of this validation, we find a very convincing agreement between measurement and prediction of the developed model framework.

**Table 1:** Comparison of supersaturation rates for all characteristic experiments. The parameters used for each characteristic experiment are also indicated in the table. An upward pointing arrow was added to parameters that correlate positively with the supersaturation rate, while parameters that correlate negatively with the supersaturation rate are succeeded by a downward pointing arrow. Parameters that exhibit an optimum (and therefore have a correlation coefficient of 0) are written without arrow. The constant parameters were the temperature of 22 °C for all systems and the dry film thicknesses of MAPI of 1.2 µm and 0.9 µm of double cation (See Note S8, Supporting Information for details).

Quenching Method	Sample width/Position x [mm]	Nozzle Width D[mm]	Height h[mm]	Velocity $u_{0}[ms^{-1}]$	Add. Parameters	Supersat. Rate MAPI [s^−1^]	Supersat. Rate 2cat [s^−1^]
Vacuum	32 ↘	n.a.	n.a.	$5.4\times \;10^{-0.094s^{-1}\times t}↗$	*k*_p_ = 0.54 *s* ^−1^ ↗	1.7 × 10^−1^	2.9 × 10^−2^
Gas (stat.)	32↘	1↘	3↘	100↗	–	3.0 × 10^−3^	6.1 × 10^−5^
Gas (dyn.)	MAPI: (5s^−1^) × *t*, 2Cat: (0.5s^−1^) × *t*	0.1↘	3↘	100↗	*L* = 15 cm, θ = 90°	2.5 × 10^0^	7.4 × 10^−2^
Antisolvent	32↘	1↘	3↘	0.1↗	*q*_c,CB, MAPI_ = 2.9 × 10^3^ mol m^−3^ ↗ *q*_c,CB, 2cat_ = 3.6 × 10^3^ mol m^−3^ ↗	9.4 × 10^1^	1.7 × 10^2^

#### 2.2.1 Vacuum Quenching with Interferometric In Situ Characterization

Vacuum quenching is an established processing method to realize high supersaturation rates on large areas,^[^ ^8^, ^15^, ^26^, ^63^, ^64^, ^65^ ^]^ however due to the sample handling it has only been applied with batch‐to‐batch processing to our knowledge. Though widely used in literature as well as in this work, the term “vacuum quenching” is misleading. The reason is that “vacuum quenching” insinuates that evaporation of solvent into a (near) vacuum, which is in essence boiling of solvent, would be the main involved mass transfer process. However, vacuum quenching commonly starts with an antechamber filled with air at atmospheric pressure that is pumped out gradually. Therefore, at the beginning of the quenching process, the drying dynamics are governed by an air flow introduced by the suction of air out of the antechamber. A more suitable name would therefore be “gas quenching at reducing pressure” or “vacuum‐assisted gas quenching” as our team prefers to call it.^[^ ^8^, ^15^ ^]^ The educated reader might object that, at very low pressures, the mass transfer dynamics could fundamentally change. However, we present a very detailed discussion in Note S5 (Supporting Information) finding that, opposite to intuition, boiling of solvent or other changes of mass transfer mechanisms do not occur during vacuum quenching at all. In short, nucleation of a solvent gas bubble at the liquid‐substrate interface, a precondition of boiling, is neither observed nor promoted due to surface‐energy considerations. Consequently, Equation (6) should remain valid during the entirety of the vacuum quenching process. Direct evidence supporting this hypothesis is presented in the following analysis by measurements of the thickness decrease of perovskite solution films (we started from general knowledge and did not perform any fitting for the complete drying of the MAPI solution and most of the drying process of the double cation solution).

As the characteristic experiment, we consider a standard, rectangular vacuum chamber with volume *V* connected to a vacuum pump on one side (with an optional supply of fresh drying gas on the left side), as depicted in **Figure** **2**. The substrate coated with perovskite solution is placed in the antechamber that is initially at atmospheric pressure *p*_atm_ and, subsequently, the air is extracted with a constant pump rate S_out_ = Δ*V* / Δ*t*. As a consequence, the pressure in the chamber will drop approximately as

(10) $$p\;\left(t\right)=\left(p_{\mathrm{atm}}-p_{\mathrm{stab}.}\right)e^{-k_{p}\cdot t}+p_{\mathrm{stab}.}$$

where *p*_stab._ is the minimum reachable pressure (given the leak rate of the system), and *k*_p_ is the rate of pressure decay (we neglect the impact of the evaporation of solvent molecules on the chamber pressure). By measurement of static pressure, we find for our vacuum system *k*_p_ = 0.55 s^−1^ as explained in Note S6 (Supporting Information) (we added an empirical linear term of pressure decrease). As a consequence, an air flow is created in the chamber, which we simulate in great detail with Ansys Fluent 2023 R2. To achieve this, we replicate the used vacuum chamber exactly with a CAD model and simulate the air flow with the Ansys k‐ε 3D solver (details in Note S6, Supporting Information) taking as a boundary condition the flow rate causing the observed pressure decay. We extract the evolution of the gas velocity *u*_0_(*t*) at ≈2 cm over the sample from this simulation. Consistently, we find that after a short time (<0.05s) the gas flow velocity stabilizes at around *u*_0,start_ = 5.4 m s^−1^, and then decays exponentially over the substrate as

(11) $$u_{0}\;\left(t\right)=u_{0,\mathrm{start}}\;e^{-k_{u}\cdot t}$$

However, this simulated decay of air flow is considerably slower than the pressure decay (*k_u_* = 0.095 s^−1^). We attribute this to accelerated pressure decay due to the fast equilibration of pressure within the system when the valve is opened (occurring close to the speed of sound), while stabilized, subsonic flow out of the main chamber as simulated by Ansys is much slower. Although it remains unclear whether the applied Ansys simulation toolkit is the appropriate method for predicting air flow under these extreme conditions, the significant difference between *k*_p_ and *k*_u_ is pivotal for predicting the drying dynamics during vacuum quenching. From an experimental point of view, we consider this difference very plausible because a large dead volume of already pumped‐down pipe (≈4 m length) is connected to the chamber, enabling a fast decrease of pressure when the valve close to the chamber is opened. Contrarily, the air flow is caused by the continuous action of the pump (until the leak rate equals the pump rate) and therefore decays much slower. For practical use of our models, we would highly recommend either directly measuring the air velocity over time within the chamber (accounting for the decreasing pressure^[^ ^66^ ^]^) or measuring the drying dynamics of a simple reference, for example, pure solvent film, and then use *k_u_* and *u*_0,start_ as free fit parameters (we have not done this herein because we prefer model validation from general principles to pure mathematical fitting of parameters).

The complexity of vacuum quenching lies in the correct prediction of β_ig_(*t*) in Equation (6). We have $\beta_{\mathrm{ig}}\propto p{\left(t\right)}^{n-1}\cdot u_{0}^{0.5}$ due to the Sherwood correlation for laminar air flow (see Note S2, Supporting Information for details), where typically *n* = 0.33 for a laminar air flow and *n* = 0.45 for turbulent air flow. As *p*(*t*) and *u*_0_(*t*) decay with time exponentially, assuming a laminar air flow, β_ig_ increases exponentially as

(12) $$\beta_{\mathrm{ig}}\left(t\right)\propto e^{\left(0.77k_{p}-0.5k_{u}\right)t}$$

The above relation highlights why the faster pressure decay (as compared to the decay of air flow) has such a decisive effect on the drying dynamics. We note that *t* = 0 is the point in time when the chamber is opened and the air flow has stabilized, so possible pre‐drying needs to be taken into account. This can be an issue for the scaling of the technique, where coating takes a long time, but we assumed here no pre‐drying due to the high boiling points of the solvents.

**Figure 3:**
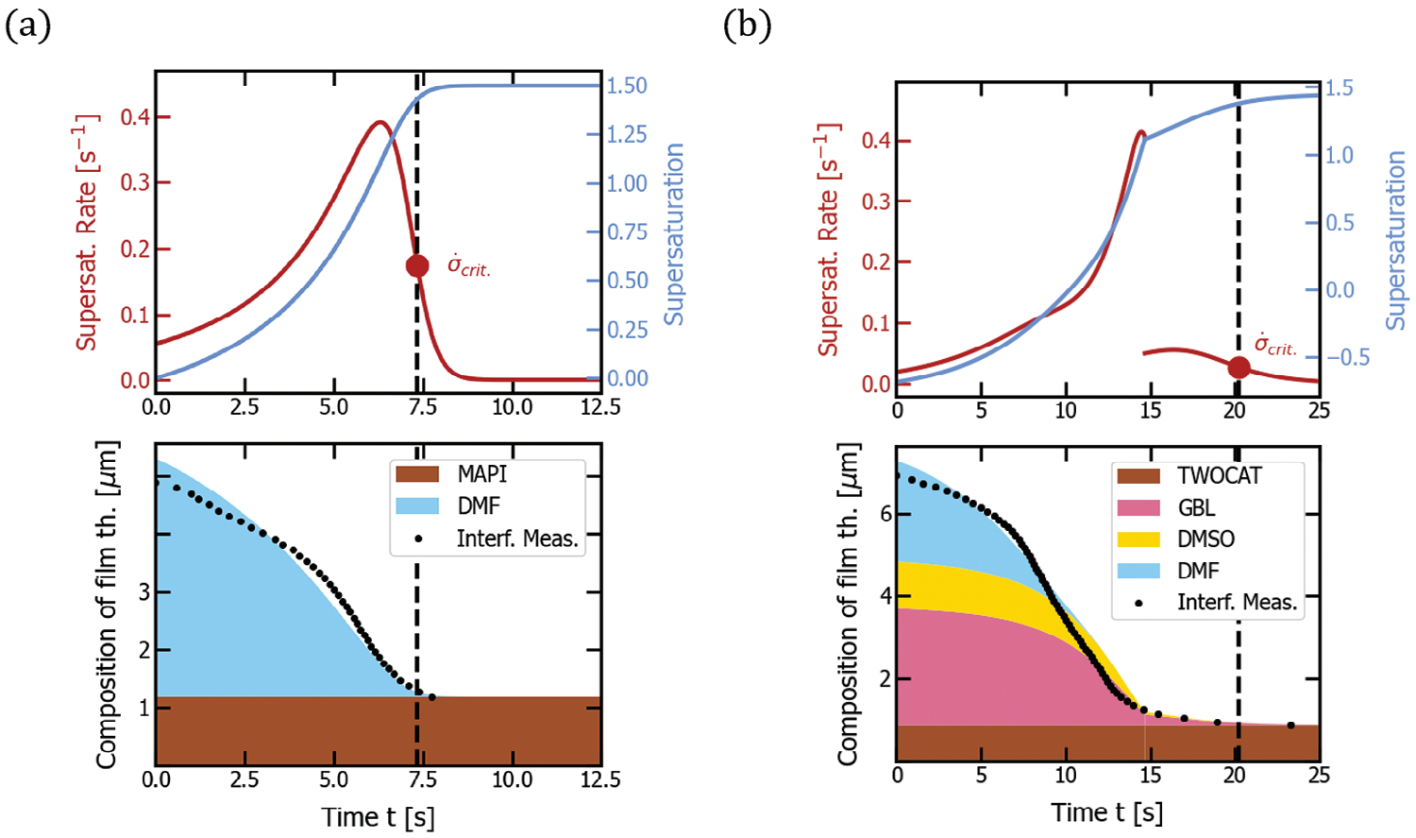
Vacuum quenching of MAPI (a) and double cation precursor solutions (b). The respective top plot shows supersaturation (light blue) and supersaturation rate (dark red) over time with a marker of critical supersaturation rate ${\dot{\sigma }}_{\mathrm{crit}.}$ reached at the time indicated by a dashed line. The respective bottom plot depicts the thickness composition of the film over time (*d*_1_,…, *d_n_*, *d*_pvk_), that is how much of the film thickness is comprised of which material, with added data from interferometric measurements (black dots). The corresponding experimental parameters are noted in Table 1. The initial, exponential increase in drying rate is caused by the fast decay of chamber pressure, increasing the mass transfer coefficient, when the valve to the already pumped‐down pipe is opened. The double cation solution experiences a deceleration of drying in the end of the drying process, which can likely be explained by the formation of an intermediate phase reducing the solvent activities.

In order to test the above assumptions, we performed interferometric measurements on the drying thin‐films in our vacuum chamber (see Figure 2; Note S3, Supporting Information). The resulting dynamic thickness data are overlaid as black dots with the simulation results in **Figure** **3**. Notably, the MAPI solution shows good correspondence with the model at unit solvent activity, consistent with our previous studies.^[^ ^20^, ^22^, ^41^ ^]^ The supersaturation peaks just before the critical concentration is reached (compare the dark red line in the top plot of Figure 3a with the point of critical supersaturation rate indicated by the red dot and the dashed black line in the same Figure). When introducing solvents with higher boiling points and a second drying regime with reduced solvent activities (γ_DMSO_ = 0.16, γ_GBL_ = 0.11), the drying rate in the double cation solution is consistently decreased and decelerated just before the crystallization. While supersaturation increases monotonously for both cases from ≈0.5 to 1.5 (see light blue solid lines in Figure 3), the calculated supersaturation rate has a discontinuity in the double cation solution when the drying regime changes abruptly (dark red line at the right side of Figure 3). This is clearly not physical. As mentioned above, this issue could be resolved in the future by the use of a mixture model for double‐cation perovskite, describing the solution activity as a function of concentration. At the current state, it is however omitted to avoid overfitting. Finally, we determine ${\dot{\sigma }}_{\mathrm{crit}.}=0.17s^{-1}$ for the MAPI solution and ${\dot{\sigma }}_{\mathrm{crit}.}=0.029\;s^{-1}$ for the double cation solution. This difference is caused by the use of higher boiling point solvents and the activity reduction in the double cation precursor solution. In conclusion, we obtained convincing evidence that the model assumptions were reasonable and that the fit of the drying dynamics is accurate for the double cation precursor solution, as well. In particular, the hypothesis of different decay times of pressure and air flow gives rise to a characteristic shape of the drying curve that reproduces the data quite accurately (if pressure and air flow decay were completely in sync, the initial shoulder in the drying dynamics would be hardly visible and the drying process would be much slower).

For the practical application of the model, the impact of all process parameters can now be determined. The sample width, *x*, and *k*_u_ correlate negatively with ${\dot{\sigma }}_{\mathrm{crit}.}$, while *k_p_* and *u*_0,start_ correlate positively with ${\dot{\sigma }}_{\mathrm{crit}.}$ (see arrows in Table 1). Conclusively, an antechamber and the connected pumping system must be designed in a way to quickly reduce static pressure while still maintaining high air flow. Furthermore, the substrate size can be an issue (even if vacuum quenching is more scalable than static gas quenching and antisolvent quenching as depicted in Figure S18, Supporting Information). These predictions are perfectly in line with empirical experience.^[^ ^26^, ^64^, ^65^, ^67^ ^]^ They also explain conclusively why an intentional leak of defined size can be beneficial for vacuum quenching of large‐area perovskite substrates, highlighting the excellent prediction capability of the model.

#### 2.2.2 Gas Quenching on Static and Moving Substrates

**Figure 4:**
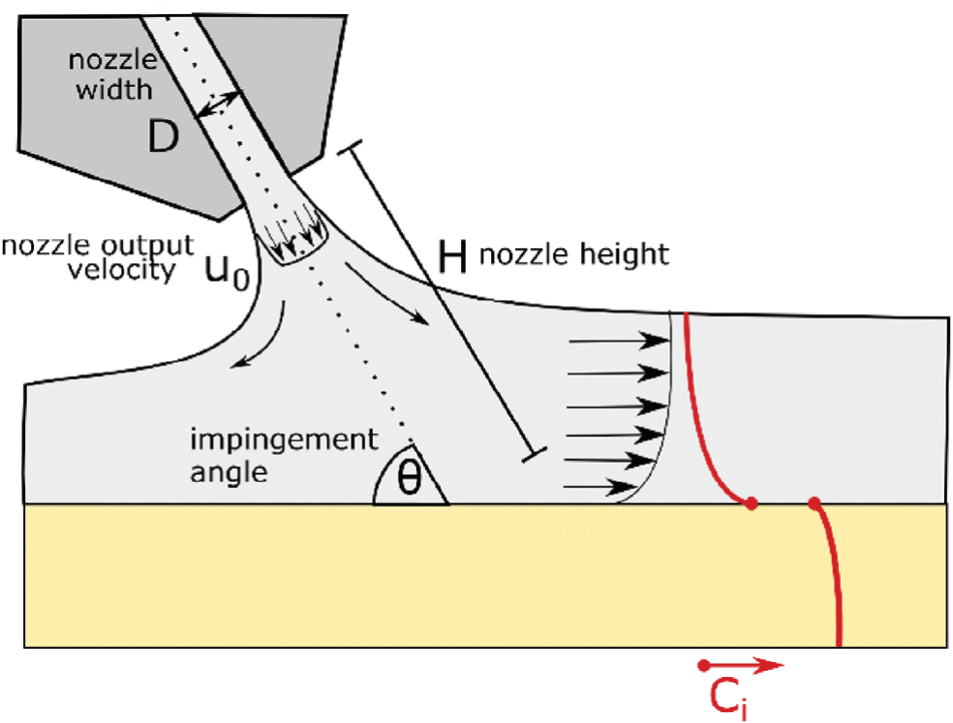
Gas quenching setup: An air nozzle of width D (round or slot) is placed over the solution film at height, *h*, in a mounting angle θ, ejecting gas out of the nozzle outlet with velocity *u*_0_. The concentration of solvent, *c*_i_ , falls off toward the film surface (this gradient is approximated as zero herein). The solvent is then present in the gas phase with a certain concentration of gaseous solvent. The concentration jump at the phase boundary can be described by Raoult's law. The gaseous solvent is transported into the gas phase until the edge of the boundary layer is reached.

In gas quenching, the perovskite solution film is dried rapidly by forced convection of a certain gas (nitrogen or dry air) over the substrate. The effectiveness of gas quenching depends heavily on the chosen air flow geometry and only slightly on the properties of the gas used for drying (this is due to the very similar properties of different molecules in gaseous form acting as ideal gases). In order to identify all critical parameters for gas quenching, we view the setup shown in **Figure** **4**. The quenching gas is ejected with velocity, *u*_0_, (as measured by a mass flow controller) from a nozzle of width, *D*, mounted at a height of *H* with an angle θ over the substrate. Additionally, the nozzle geometry must be specified as a round nozzle or slot nozzle (there is also the possibility of using nozzle fields^[^ ^39^ ^]^). These experimental parameters allow for the use of common correlations for predicting the local mass transfer coefficient, β_ig_, of gaseous solvent *i* into the gas phase (see Note S2, Supporting Information). Therefore, with exactly these parameters at hand, comparability of the setups can be enforced (additional parameters such as the gas pressure are neither needed nor helpful because they depend on the other specified parameters in a complex fashion.).

**Figure 5:**
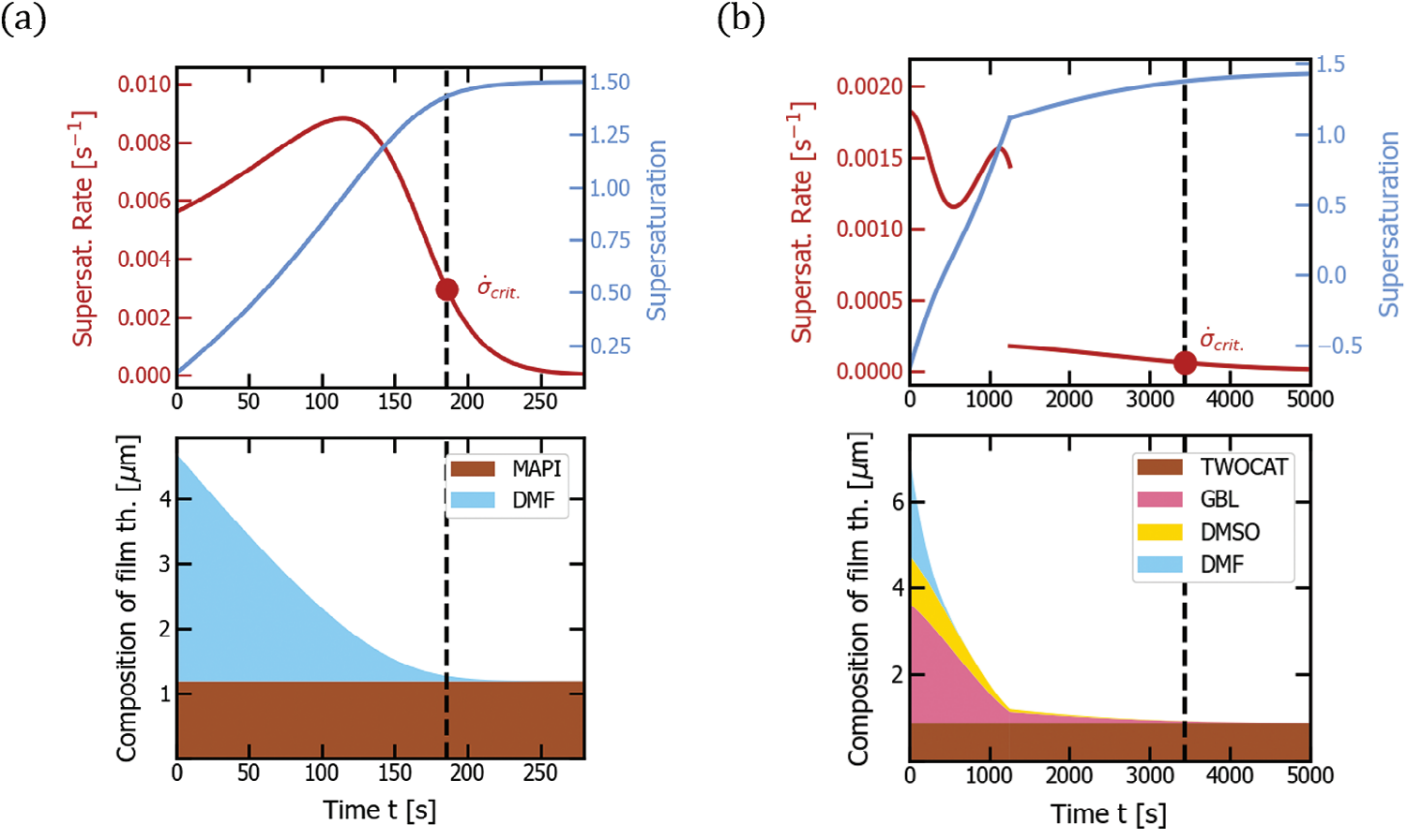
Gas quenching of MAPI (a) and double cation precursor solutions (b). The respective top plot shows supersaturation (light blue) and supersaturation rate (dark red) over time with a marker of critical supersaturation rate ${\dot{\sigma }}_{\mathrm{crit}.}$ reached at the time indicated by a dashed line. The respective bottom plot depicts the thickness composition of the film over time (*d*_1_,…, *d_n_*, *d*_pvk_), that is how much of the film thickness is comprised of which material. The corresponding experimental parameters follow in Table 1. The double‐cation solution experiences a deceleration of drying at the end of the drying process, which can likely be explained by the formation of an intermediate phase.

In static gas quenching, a compromise between the magnitude and homogeneity of β_ig_ must be made. As the air speed increases and the distance to the sample decreases, higher values of β_ig_ can be achieved, but this is only true for a very small region right in the center below the nozzle^[^ ^20^ ^]^ (see black line in Figures S11 and S12, Supporting Information). In other words, increased lateral homogeneity of β_ig_ comes at the expense of lower magnitude of β_ig_, which in turn decreases the achievable supersaturation rate [see red and blue lines in Figures S11,S12, and Figure S18 (Supporting Information) showing the decay in supersaturation rate with increasing substrate size]. The characteristic experiment for static gas quenching is parameterized in accordance with a typical high‐pressure air gun used during spin coating^[^ ^68^ ^]^ (more details in Experimental Section). **Figure** **5** shows the corresponding dynamic simulation results for the MAPI solution and the double cation solution, which we validated by measurements of thickness decrease upon evaporation in earlier works.^[^ ^20^, ^22^ ^]^ As detailed as well in our previous work,^[^ ^22^ ^]^ the MAPI precursor solution dries with an almost constant slope until the end of the drying process where it experienced an exponential deceleration (Figure 3). Critical supersaturation (0.003 s^−1^) is reached at the end of the drying process (the red point on the left side graph of Figure 5). When introducing solvents with higher boiling points and a second drying regime with reduced solvent activity (γ_DMSO_ = 0.16, γ_GBL_ = 0.11), the reachable supersaturation rate is decreased by about one order of magnitude (6.1 · 10^−5^ s^−1^) for the double cation precursor solution (the red point at the right side graph of Figure 5). As mentioned in the last section, the same discontinuity occurs in the supersaturation rate due to the simplicity of the model (dark red line in of Figure 5b).

**Figure 6:**
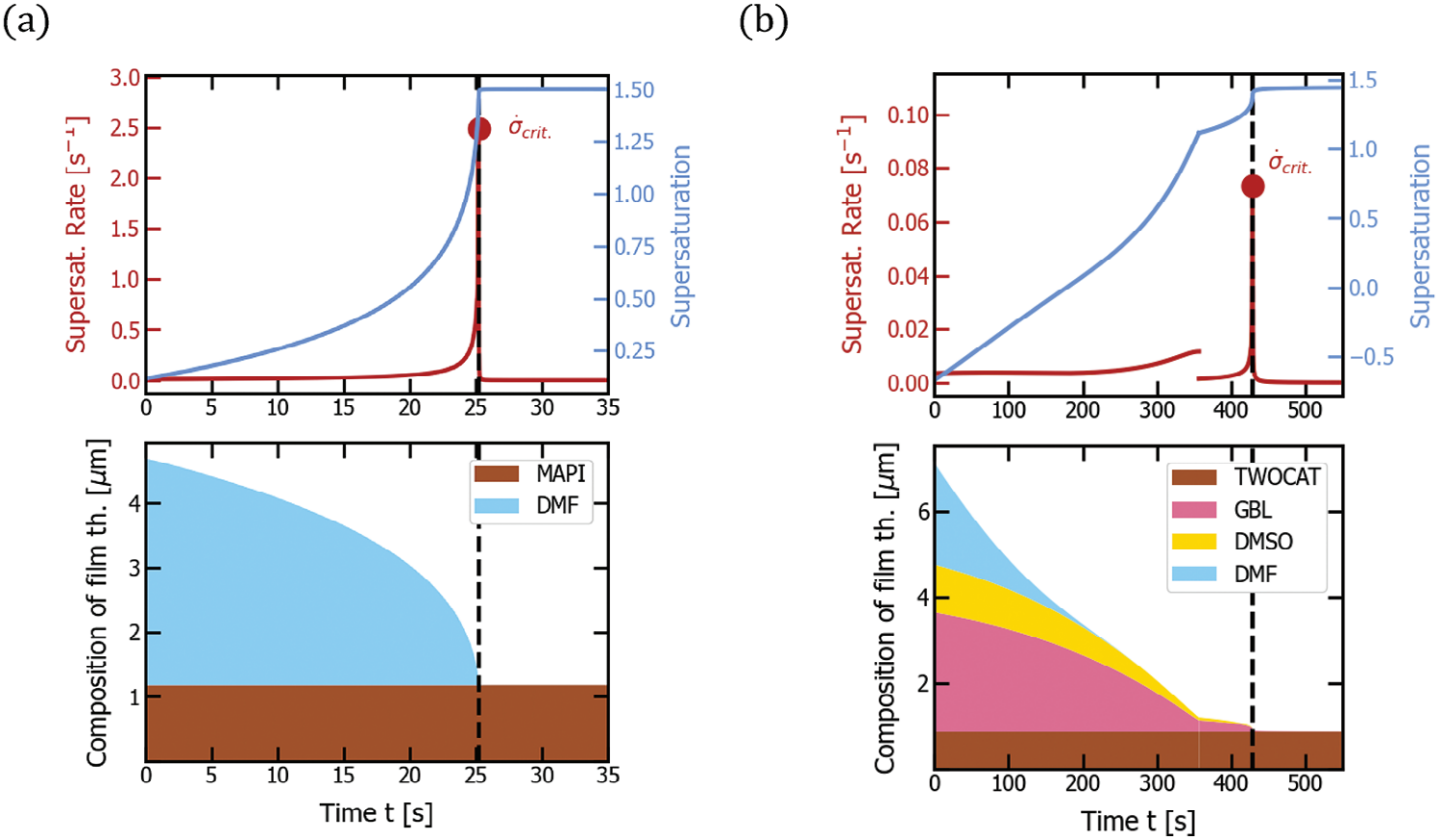
Dynamic Gas quenching of MAPI (a) and double cation precursor solutions (b). The respective top plot shows supersaturation (light blue) and supersaturation rate (dark red) over time with a marker of critical supersaturation rate ${\dot{\sigma }}_{\mathrm{crit}.}$ reached at the time indicated by a dashed line. The respective bottom plot depicts the thickness composition of the film over time (*d*_1_,…, *d_n_*, *d*_pvk_), that is how much of the film thickness is comprised of which material. The corresponding experimental parameters follow in Table 1. The double‐cation solution experiences a deceleration of drying at the end of the drying process, which can likely be explained by the formation of an intermediate phase.

Building on the static gas quenching case, we develop the concept for dynamic gas quenching as it corresponds to static gas quenching with a linearly moving substrate. Assuming linear lateral movement with a constant velocity *v*, the dynamic case simply needs to consider a temporally varying mass transfer coefficient,

(13) $$\beta_{\mathrm{ig}}\left(x\left(t\right)\right)=\beta_{\mathrm{ig}}\left(v\times t\right)$$

where β_ig_(*x*) is the spatially inhomogeneous mass transfer coefficient (with an assumed nozzle position *L*[m]) varying with the dimension *x*[m], *v*[m s^−1^] is the web speed and *t*[s] is the time. As we showed before,^[^ ^20^ ^]^ by leveraging the lateral movement of the substrate, the issues with the inhomogeneity of the mass transfer coefficient described above can be mitigated. This is achieved by timing the drying process in a way that the film crosses critical concentration always exactly under the nozzle center at the maximum of local mass transfer (see dynamics in **Figure** **6**). Therefore, we can reduce the nozzle width from 1 to 0.1 mm and still fabricate a homogeneous perovskite film at higher supersaturation rates (we assume the high‐pressure gas stream does not deform the solution thin film or that it is only deformed for a short period of time).

Like in static gas quenching, the ratio of vapor pressures of the solvents as well as their activities have a large impact on the supersaturation rate. For the chosen parameters, the MAPI solution reaches again one order of magnitude higher values in critical supersaturation rate (2.5 s^−1^) than the double cation precursor (0.07 s^−1^). It is important to stress again that these high values are only reached in the case where the web speed is fine‐tuned to an exact value, enabling that critical concentration is reached right under the nozzle center. Experimentally, this is challenging to demonstrate if the wet film thickness is not constant.^[^ ^52^ ^]^ In particular, since the MAPI solution dries very fast, optimal tuning will be very hard to control, causing the effective supersaturation rate much lower than reported above. In this context it is worth noting that the models can be used to predict (experimentally tested) process windows of high enough supersaturation, giving explicit guidelines to experimentalists.^[^ ^20^ ^]^ Furthermore, we directly observed the correlation between the position of the crystallization onset (determining the reachable supersaturation rate) and the morphology of slot‐die coated perovskite thin films.^[^ ^52^ ^]^

For the practical application of the model, the impact of all process parameters can now be determined. The sample width, *x*, the slot width, *D*, and nozzle height, *h*, correlate negatively with ${\dot{\sigma }}_{\mathrm{crit}.}$ (see arrows in Table 1), while *u*_0_ correlates positively with ${\dot{\sigma }}_{\mathrm{crit}.}$. Conclusively, gas quenching benefits from using very narrow nozzles that are very close to the substrate, which is consistent with the experimental observation.^[^ ^20^ ^]^ However, for static gas quenching, lateral homogeneity of ${\dot{\sigma }}_{\mathrm{crit}.}$ can be an issue limiting the choice of process parameters, which can be partially mitigated by fine‐tuning dynamic gas quenching, as explained above. Furthermore, the gas speed is limited by the viscosity of the film, as velocities over 150 m s^−1^ can easily deform the wet film irreversibly.^[^ ^20^ ^]^ Overall, the model is in line with experimental observation.

#### 2.2.3 Antisolvent Quenching

**Figure 7:**
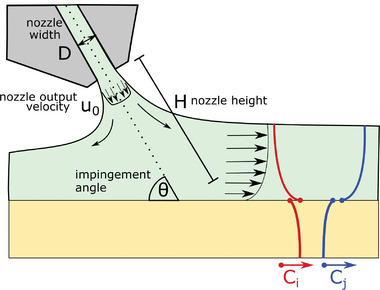
Antisolvent quenching setup: An air nozzle of width D (round or slot) is placed over the solution film with height, *h*, in a mounting angle θ, ejecting an antisolvent with outlet velocity *u*_0_. Subsequently, the concentration of solvent, *c_i_* , inside the film volume element (yellow) will decrease, while increasing outside of the film as determined by the flow profile. The inverse relation is true for the antisolvent concentration that increases inside of the film. Here, we assume zero concentration gradients inside of the film and model the concentration gradients outside of the film by the mass transfer coefficient. The respective concentration jumps at the film surface are caused by the perovskite precursor chemicals that are assumed not to diffuse into the top volume element.

Regarding **Figure** **7**, it becomes apparent that antisolvent quenching has an astonishingly high degree of commonality with gas quenching. Instead of a gas, a liquid antisolvent is ejected with initial flow speed, *u*_0_, from a nozzle of width *D*, height *H*, angle θ, and spreads over the deposited solution film forming a boundary layer. In common antisolvent quenching processes, the solution film dries for some time before applying the antisolvent, which needs to be considered in the model by starting from a smaller solution film with a higher concentration. As alluded to in Section 2.1.4, we assume that due to the initial drying, the perovskite solution has a much higher viscosity than the ejected antisolvent. Accordingly, we assume, the flow of antisolvent does not deform or displace the perovskite solution film. We also assume, as detailed in Section 2.1.4., a diffusion of two volume elements designated as “outside of the film” (light blue area in Figure 7) and “inside of the film” (light yellow area in Figure 7) to establish an analogy to gas quenching and use Equations ((7), (8), (9)) for calculating the mass transfer dynamics. We note that, at the current state of the work, the model is limited to a crystallization occurring while the supply of fresh antisolvent is kept alive. Given that the observed dynamics predict the crystallization to occur already after milliseconds, we suppose this is the case in most antisolvent processes. We do not consider any changes to the supersaturation by the evaporation of residual antisolvent out of the thin film, so far. The above assumptions are motivated by the fact that antisolvent quenching can be successfully performed with a large variety of antisolvents, all having different boiling points.^[^ ^28^ ^]^

**Figure 8:**
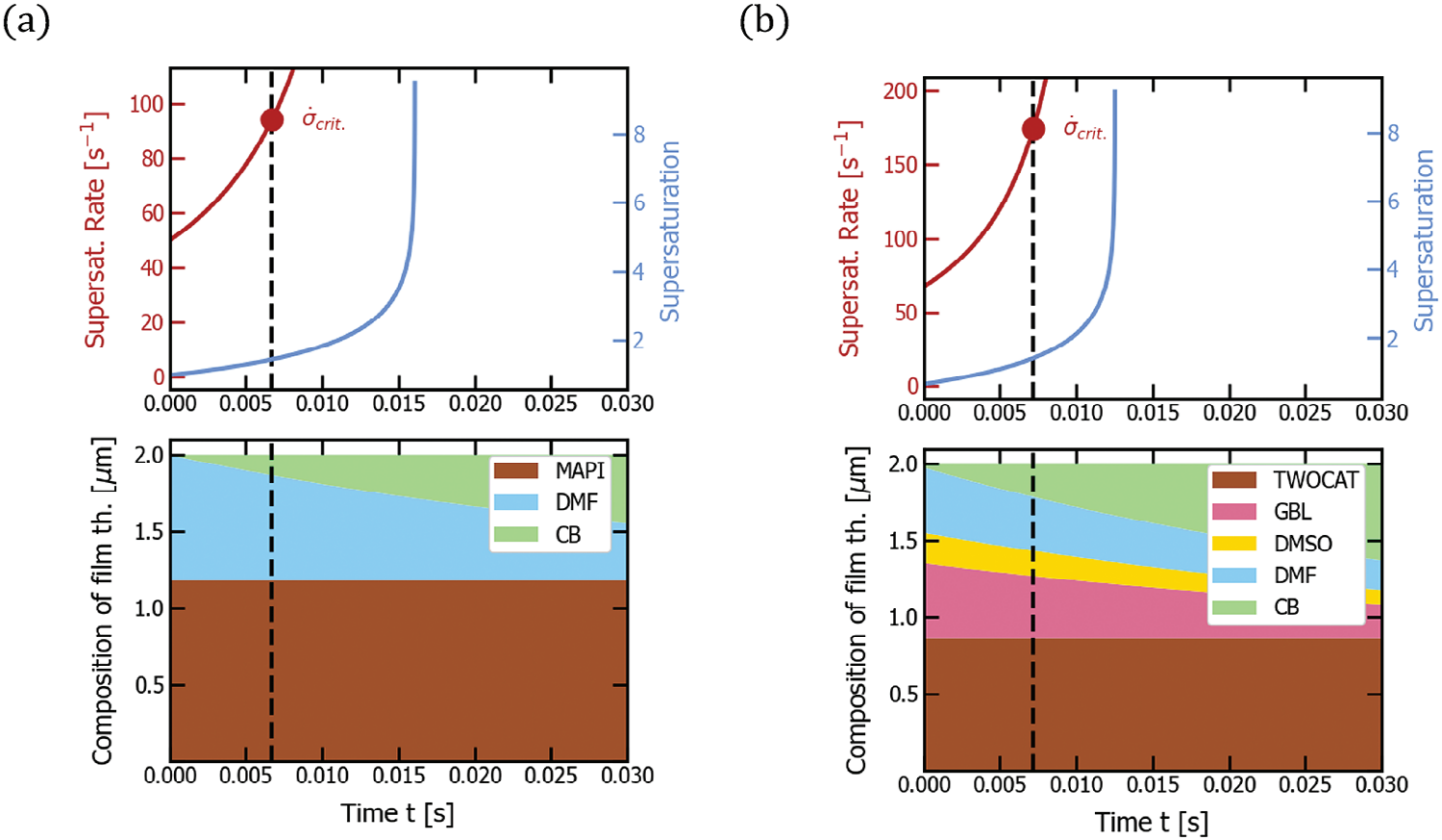
Antisolvent quenching of MAPI (a) and double cation precursor solutions (b). The respective top plot shows supersaturation (light blue) and supersaturation rate (dark red) over time with a marker of critical supersaturation rate ${\dot{\sigma }}_{\mathrm{crit}.}$ reached at the time indicated by a dashed line. The respective bottom plot depicts the thickness composition of the film over time (*d*_1_,…, *d_n_*, *d*_pvk_), that is how much of the film thickness is comprised of which material. The corresponding experimental parameters follow in Table 1. Due to the reduction in solubility by antisolvent diffusion, the crystallization happens much earlier than in the previously shown quenching techniques.

Importantly, antisolvent quenching outranks the other quenching methods in terms of supersaturation rate by at least two orders of magnitude (see **Figure** **8** and Table 1): We find a supersaturation rate of 94 s^−1^ for MAPI and 174 s^−1^ for double cation. The reason for these high values is the much faster mass transfer over the liquid–liquid interface as compared to a liquid–gas interface, where the solvent evaporates reducing its density by around three orders of magnitude (for instance, DMF has a liquid, molar density of 12.9 mmol cm^−3^ and in gaseous form, as an ideal gas, 0.046 mmol cm^−3^). The high supersaturation rate for the double cation solution explains the very widespread use of antisolvent quenching in the prototyping of solution‐processed PSCs as well as the difficulties to transfer antisolvent processes to gas or vacuum quenching. Furthermore, since the diffusion coefficients of the common, liquid solvents are fairly similar, there is almost no impact of the solution formulation on the supersaturation rate (of course, the accuracy of the model depends on the correct estimation of diffusion coefficients in the solution mixture. Here, we used a simple Tyn/Calus liquid–liquid diffusion^[^ ^39^ ^]^). It must be noted that also in liquid–liquid diffusion, activity changes due to interactions of solvent and solute can occur, which is not yet included in the model. At this point, we do not make more precise statements because liquid–liquid diffusion cannot be investigated by interferometry given that the refractive indices of all solvents are very similar.

For the practical application of the model, the impact of all process parameters can now be determined. The sample width, *x*, the slot width, *D*, and nozzle height, *h*, correlate negatively with ${\dot{\sigma }}_{\mathrm{crit}.}$ (see arrows in Table 1), while *u*_0_ correlates positively with ${\dot{\sigma }}_{\mathrm{crit}.}$ Due to the fast liquid‐liquid mass transfer as compared to liquid–gas mass transfer, the process parameters are however less important in antisolvent quenching than in the other quenching methods due to the consistent yield of very high ${\dot{\sigma }}_{\mathrm{crit}.}$. Remarkably, this model prediction matches the experimental observation because the antisolvent is successful for a broad range of *u*_0_ once solubility issues are eliminated^[^ ^28^ ^]^ and other process parameters are commonly not well controlled either during manual processing. Furthermore, even if the scaling of the method is not ideal, supersaturation rates are consistently above the ones of concurrent methods for all substrate sizes (see Figure S18, Supporting Information).

The parameter *q*_C_ leads to the main differences in the mass transfer dynamics when different antisolvents are used. In particular, for our MAPI and double‐cation solutions, we determined a relative increase in *q*_C_ of ≈25% for the double cation (see Figure S2, Supporting Information). It is however to be expected that this parameter is a function of the used solvents as well as the stoichiometry of the perovskite. While this analysis is based on two reference systems only, we highly recommend each group apply our models to determine the parameter *q_C_* for their specific solution and/or perovskite composition, according to the simple experiment described in Note S1 (Supporting Information). Another important parameter is the viscosity whose impact we analyze in Figure S19 (Supporting Information). When using very viscous solvents, rheological film deformation of the perovskite layer can become an issue (which is not considered in the model). Furthermore, the steady supply of fresh antisolvent during quenching, which is one of the preconditions for fast diffusion, can be a challenge in industrial fabrication processes, where solvent usage should be limited.

### 2.3 Comparison and Implication of Results

Comparing the supersaturation rates obtained from the modeling framework in Table 1, we can extract valuable information about the suitability of either quenching method. As explained above, quenching effectiveness can be benchmarked directly in supersaturation rates. The higher the supersaturation rate, the more likely smooth perovskite films are achieved.^[^ ^20^, ^22^, ^50^, ^51^, ^52^ ^]^ Depending on the exact crystallization dynamics (and all solution parameters that affect these), different thresholds in supersaturation rate may be needed.^[^ ^69^, ^70^ ^]^ In earlier works,^[^ ^22^ ^]^ we showed that MAPI perovskite can crystallize in a smooth film when using static gas quenching. So, supersaturation rates of 10^−3^ s^−1^ are sufficient for MAPI crystallization if certain additives are used.^[^ ^18^, ^20^, ^22^ ^]^ Comparing the quenching dynamics of MAPI with double cation, we consistently find a difference of about one order of magnitude in supersaturation rate due to the suspected formation of intermediate phases and the use of higher boiling point solvents.^[^ ^41^ ^]^ This difference explains the much greater challenges that researchers and technologists face when processing this solution formulation with static gas quenching, strongly encouraging the use of vacuum or dynamic gas quenching^[^ ^20^ ^]^ to reach comparable supersaturation rates as for MAPI (we estimate that ≈10^−2^ s^−1^ are needed for processing a smooth double cation film).

Remarkably, for vacuum quenching, the difference in supersaturation rate between the MAPI and the double cation solutions is just a factor of five (as compared to more than 30 for the gas quenching techniques), which is due to the exponential acceleration of the drying dynamics. This greatly simplifies process transfer from one solution system to another, which is in line with the recent popularity of vacuum quenching for quenching FAI‐based perovskites.^[^ ^35^ ^]^ Considering in addition the rather homogeneous distribution of mass transfer coefficients in laminar air flows, vacuum quenching offers minimal scaling losses of supersaturation rate. A clear drawback of vacuum quenching is however the complexity of the required apparatus restricting its use to batch‐to‐batch processing. Furthermore, it might be difficult to scale the technique in an economical way, while still maintaining fast enough air extraction and avoiding accumulation of solvent vapor in the chamber. Gas quenching has, in comparison to vacuum quenching, a much simpler setup. Static gas quenching reaches however, in contrast to vacuum quenching, insufficient supersaturation rates, and additionally, exhibits poor scaling behavior due to the inhomogeneity of mass transfer. These disadvantages can be partially mitigated by introducing dynamic gas quenching, keeping the crystallization onset in the center of high mass transfer under the gas nozzle. In this way, even higher supersaturation rates than in vacuum gas quenching can be reached. However, it can be challenging to achieve the needed fine‐tuning of web and gas speeds to maintain this condition–especially when the wet film thickness is not constant. Lastly, antisolvent quenching reaches consistently very high supersaturation rates, outranking the other quenching techniques by at least two orders of magnitude in supersaturation rate. This great effectiveness decreases the importance of the exact values of the process parameters, since reached supersaturation rates are always sufficiently high. Therefore, antisolvent quenching is a robust technique for prototyping new material systems. However, drawbacks are the increase of solvent use during fabrication having negative impacts on sustainability and cost‐effectiveness. Furthermore, the additional complexity of processing by introducing a new solvent increases the probability of failure modes.

Most importantly, the above‐described methodology of quantifying supersaturation rates enables the transfer from one quenching process to another. By calculating and comparing supersaturation rates, it becomes possible to estimate the effectiveness of a newly designed quenching process and predict the necessary experimental parameters. If researchers and technologists always report supersaturation rates along with their experiments for reference, the design of drying and coating machines can be significantly simplified. We strongly encourage the perovskite research community to apply and further improve the modeling framework. This is facilitated by the full disclosure of the Python codes as well as the strict, modular design of the software. We firmly believe that for the design of future large‐scale perovskite pilot lines involving major capital investment, the understanding and prediction capability of quenching methods is of utmost importance. In this way, the art of fabricating perovskite solar cells comes one step closer to a testable, reproducible engineering science.

## 3 Conclusion

In this work, we present three main novelties: 1) a quantitative modeling framework of mass transfer dynamics for comparing all common perovskite quenching processes (Vacuum, Static Gas, Dynamic Gas and Antisolvent), released as an open source, an extensible software package called SupersatRN‐C. 2) A deep understanding of the impact of every relevant process and material parameter on the modeled mass transfer dynamics (see arrows in Table 1), which we showcase by presenting real‐word, characteristic experiments, 3) the introduction of supersaturation rates as a decisive benchmark for all common quenching methods (presented for the characteristic experiments in Table 1). For vacuum quenching, we test the predicted drying dynamics with interferometric measurements in an existing chamber and simulate the gas speed evolution over the substrate with Ansys Fluent 2023 R2. For static and dynamic gas quenching, we tested these dynamics in earlier works^[^ ^20^, ^22^ ^]^ by a similar approach (here, we directly measured the air flow speed with a hot wire anemometer instead). Our results are the first of its kind quantitative mass transfer analysis that offers a unified description of different quenching methods. By fully disclosing the codes of our simulation framework, we aim at maximizing its adoption not only for describing perovskite processes more accurately but also for estimating the effectiveness of large‐scaling quenching tools still on the drawing board. In the future, the models could also be used to feedback control the perovskite formation process by predicting the outcome of the perovskite deposition.

## 4 Experimental Section

### The Investigated Perovskite Solutions

The two different perovskite solution systems were investigated in great detail in this report. Methylammonium lead iodide, MaPbI_3_, (MAPI) dissolved in N,N.Dimethylformamide (DMF) with the molality 1 mmol mL^−1^ (1 m), due to the simplicity of the solution system, and double cation perovskite, Cs_0.17_FA_0.83_Pb(I_0.91_Br_0.09_)_3_, dissolved in a 4:1 (v:v) mixture of DMF and Dimethylsulfoxide (DMSO) with the molality 1 mmol mL^−1^ (1 m) and the addition of 500 µL γ ‐Butylactone (GBL) per mL to the above solution. The solutions had thus a different molality of 1m (i) and ≈0.6m (ii). This difference in molality was chosen to compensate for differences in the deposited thin film thickness caused by different rheological properties of the solutions. The final, dry perovskite thin‐film thicknesses were still different by ≈0.3 µm (as measured by simple profilometry, see Note S8, Supporting Information).

### Description of Characteristic Experiments

This section is concerned with describing the characteristic experimental setups that were chosen to calculate the mass transport dynamics in this work, culminating in the supersaturation rates listed in Table 1. The experiments were chosen in a way to maximize the comparability of the quenching methods with each other, but still approach parameter values that were used in real‐word perovskite processing. For vacuum quenching, a typical physical vacuum chamber was used, as described in great detail in Note S3 (Supporting Information). Therefore do not specify the experiment further in this Section. For static gas quenching, a round jet ejected from an air gun with a radius of 1 mm was assumed that be held 3 mm over the sample. The gas speed of 100 m s^−1^ was extracted from an earlier work, where the volume flow of gas was measured with a mass flow controller.^[^ ^20^ ^]^ For dynamic gas quenching, a much narrower slot width of 0.1 mm was used, which is on the right order of magnitude for these types of quenching.^[^ ^20^ ^]^ (This value would lead to inhomogeneity in static gas quenching). For comparability, the height was kept fixed at 3 mm. In the antisolvent quenching, a typical pipette open for reference that had a diameter ≈1 mm was used. The speed was calculated for solvent ejection to lie ≈0.1 m s^−1^.^[^ ^28^ ^]^

### Commands Executed to Create the Displayed Figures

In this section, the detail how the main figures of this work were generated from SupersatRN‐C (available on GitHub under https://github.com/MassTransferDynamics/SupersatRN‐C). For vacuum quenching, a laminar correlation for mass transfer was initialized. The correlation was averaged over a width of 32 mm (which was motivated by the approximate of the measurement point in laser reflectometry visible in Figure S4, Supporting Information). Then, a temperature of 22 °C was set as an additional environmental parameter. A film of DMF and MAPI was created, starting at a wet thickness of 5.3 µm and a dry film thickness of 1.18 µm (see Note S8, Supporting Information). Subsequently, a “VacuumDynamics” object was created with a maximum simulation time of 12.5 s. The solution was then computed and displayed in two subplots. For the double cation films, the same correlation and environment parameters were used, but the film was composed of a complex mixture of GBL:DMSO:DMF with the volume ratios derived from Section “The Investigated Perovskite Solutions”. According to the respective measurements, different wet and dry film thicknesses were chosen. Subsequently, a “SimulationQueue” of two “VacuumDynamics” objects was initialized, one with a reduced activity according to Note S3 (Supporting Information). The simulation switches from the first to the second object at the empirically determined film thickness of 1.4 µm. For the MAPI gas quenching, the same procedure was used as above, but the correlation was switched with an “AverageRoundJet” and the dynamics with a “GasQuenchDynamics” Object. The parameters were chosen as above with adjusted simulation times as required. For the dynamic gas quenching, a different, spatially resolved correlation from one of the earlier works^[^ ^20^ ^]^ was employed, describing a narrow slot jet. By performing multiple simulation runs, the web speed was further optimized, such that supersaturation rate reaches a maximum, implying that the crystallization occurred directly under the nozzle opening. For antisolvent quenching, a “LiquidRoundJet” object was initialized with the medium of liquid Chlorobenzene. The simulation was then started as explained above.

## Conflict of Interest

The authors declare no conflict of interest.

## Supporting information

Supporting Information

## Data Availability

The data that support the findings of this study are available from the corresponding author upon reasonable request.
