# Supplementary material for: Modeling and Fundamental Dynamics of Vacuum, Gas, and Antisolvent Quenching for Scalable Perovskite Processes
Source: Adv Sci (Weinh). 2024 Feb 2;11(14):2308901. doi: 10.1002/advs.202308901 (PMC11005745; doi:10.1002/advs.202308901)
Supplement: Supplementary file 1 — Supporting Information [file ADVS-11-2308901-s001.pdf]

ADVANCED SCIENCE
Open Access

Supporting Information

for *Adv. Sci.*, DOI 10.1002/advs.202308901

Modeling and Fundamental Dynamics of Vacuum, Gas, and Antisolvent Quenching for Scalable Perovskite Processes

Simon Ternes, Felix Laufer and Ulrich W. Paetzold**

Supplementary Note 1: Determination of Equilibrium and critical concentrations

Herein, we elaborate the determination of molar equilibrium and critical solute concentrations, C_0 and $C_{crit.}$, measured by the number of perovskite unit cells per volume of solution. To estimate C_0, we way 0.1 mmol of perovskite precursor chemicals and start adding solvent in steps of 5 μl. We then stir the solution for 3 min after each step and wait if the solution appears cloudy (left vial in **Figure S1**a) or transparent (right vial in in **Figure S1**a). If the solution becomes transparent after the preceding addition of solvent, we stop the procedure and calculate

$$C_0 = \frac{0.1 \text{ mmol}}{V_{\text{total}} + 0.1\text{mmol}/\,\tilde{\rho}_{pvk}}, \qquad (S1)$$

where $V_{\text{total}}[\text{cm}^3]$ is total volume of added solvent and $\tilde{\rho}_{pvk}$ is the molar density of the perovskite. This is done both for MAPI with the solvent DMF and double cation and the solvent mixture DMF:DMSO:GBL respectively (exact volume ratios given in Section 4.1). We continue this experiment adding antisolvent in the solvent mixture by premixing different volume ratios of solvent to antisolvent and then applying the same experimental procedure with the mixture of the experiment described above. We obtain the results shown in Figure S2 and fitted Equation 5 to the obtained data (In SupersatRN-C, this equation is generalized to Equation S16, where it is also considered that multiple antisolvents or different perovskites can be present in the solution at the same time).

(a) (b)

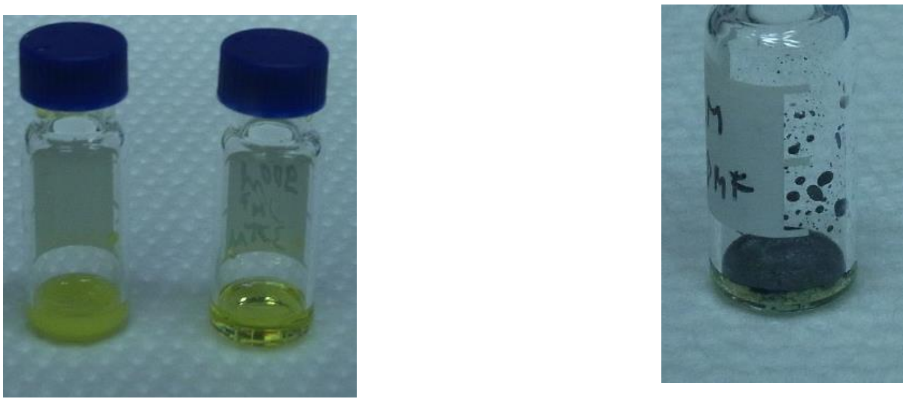

Figure S1 (a) Comparison of a not fully dissolved solution (left) and dissolved solution (right). Solvent is added in small amounts until, after 3min stirring, the solution becomes transparent. (b) Attempt to determine the critical concentration of the perovskite precursor solution. It is visible that the crystallization is incomplete on the bottom of the film. After waiting the top film will dissolve again due to the diffusion of solvent from the bottom to the top.

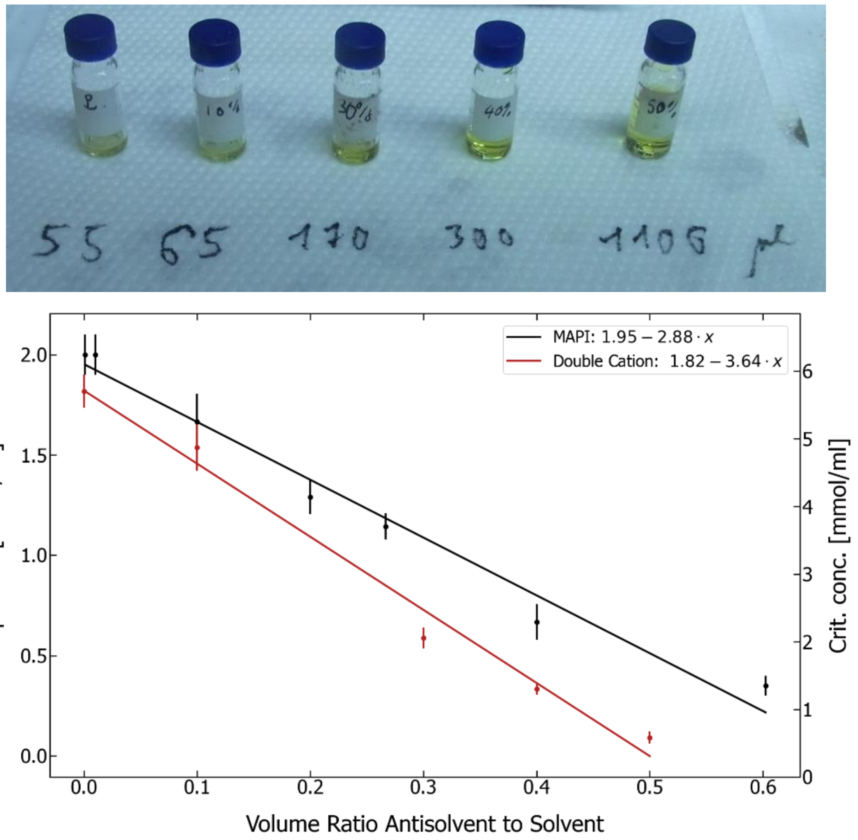

Figure S2 Experiment to determine the reduction of equilibrium concentration when adding a certain volume ratio of Antisolvent (Chlorobenzene) to the solvent mixture. Empirically, a linear relationship is observed. The critical concentration is assumed to decline with the same relationship as the equilibrium concentration, but with a constant offset (see right axis of the plot).

The determination of critical concentration is more challenging than the equilibrium concentration. The reason for this is that the crystallization of perovskite components occurs at very low solvent content, which is hard to measure with a commonly used laboratory scale. If one however attempts to increase the amount of solution, the perovskite crystallizes on the top surface of the solution first, trapping solvent in the inside due to a reduction of the diffusion coefficient in the crystallized material (see yellow bottom in vial in Figure S1b). After a certain time, the crystalline perovskite is then redissolved by diffusion von solvent from the bulk to the surface. This vertical inhomogeneity makes it very difficult to estimate the exact critical concentration (We found that only 10 μl of solvent per mmol of precursor chemicals is enough to reverse the perovskite crystallization). Therefore, we use a different method for estimating the critical concentration. For this, we consider it plausible to assume that the characteristic shape of the drying curve in a MAPI precursor is known well enough to estimate roughly which amount of solvent is approximately left in the system[1] (see Figure S3). The main difficulty with this method is that the exact wet and dry film thicknesses are unknown (the reflectometry only delivers relative thickness changes and the dry film thickness of the crystallized films is measured by profilometry only after the crystallization.). We therefore assume that the film thickness does not change during annealing (stark simplification) and we fit the drying model to the data with the open fit parameters of the wet and dry film thickness d_0 and d_∞. We further assume that the last data point is roughly recorded on the crystallization onset. With all these assumptions, we find an approximate value of critical concentration of 6340 mol m^{-3} (see Figure S3). This is done solely for the MAPI solution, because for the double cation solution, we have the additional uncertainty of activity reduction in the section drying regime (see

following section). Therefore, we assume an identical molar ratio of solvent to perovskite for the double cation solution as for the MAPI solution, and estimate the critical concentration for the double cation by renormalization with the difference in perovskite molar densities (6016 mol m^{-3}). We note that, in awareness of the uncertainty of these assumptions, we expect future works to further improve this working hypothesis. While the chosen critical concentration value impacts the quantification of supersaturation rate (and therefore must be reported with any publication of critical supersaturation rates), it does not impede relative comparability between the quenching methods (and perovskite solutions) presented in the main manuscript.

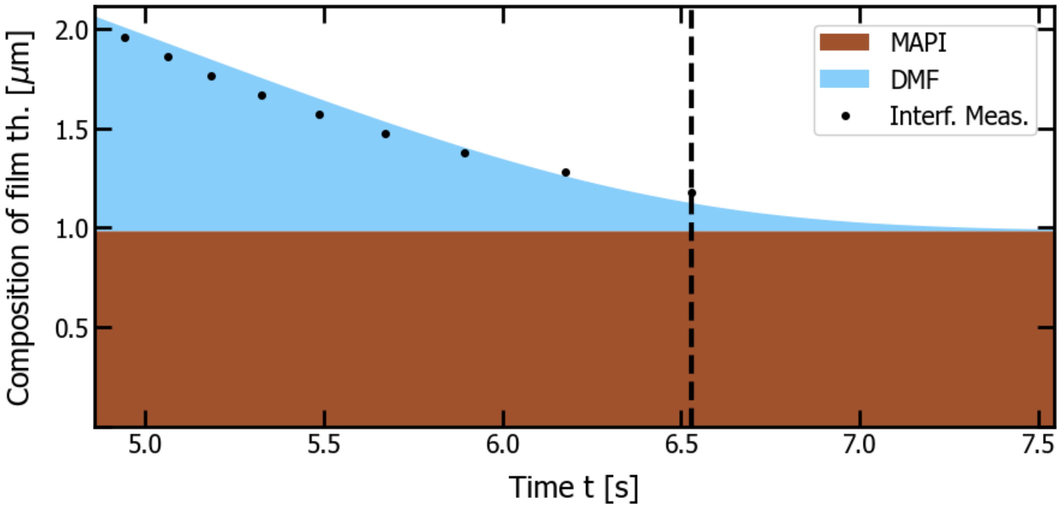

Figure S3 Experiment to estimate the concentration of the MAPI perovskite at crystallization assumed to occur at the dashed line. The drying dynamics are fitted to the interferometric measurement with the open fitting parameters of the dry film thickness and the wet film thickness. The critical concentration is then estimated by using the film thickness at this datapoint.

Supplementary Note 2: Common correlations of mass transfer

The local Sherwood number is defined as

$$\mathrm{Sh}_x = \frac{\beta_{ij} \cdot x}{\delta_{ij}} \tag{S2}$$

where x[m] is the position, δ_{ij} is the diffusion coefficient of the solvent gas i within the surrounding gas j and $\beta_{ij}(x)$ is the mass transfer coefficient[2]. It is important to note that most Sherwood correlations are expressed as an average over a certain length x, as

$$\overline{Sh}_x = \frac{1}{x} \int_0^x \mathrm{Sh}_{x'} dx' \tag{S3}$$

This can also be differentiated get the approximate, localized formulation of these correlations.

$$\mathrm{Sh}_x = \frac{d}{dx}\left(x \cdot \overline{Sh}_x\right) \tag{S4}$$

For the laminar air flow, occurring in the vacuum quenching, we use the well-known relation of average Sherwood number

$$\overline{Sh}_x = 0.664 \cdot Re_x^{1/2} \cdot Sc^{1/3} \tag{S5}$$

With the bounds of validity $\mathrm{Re} \leq 10^5$.

where $Re_x = u_0 x/\nu$ is the Reynolds number and $Sc = \nu/\delta_{ij}$ is the Schmidt number[3]. For the static gas quenching with a slot jet, we use the well-known relation as defined by Martin et al.

$$\overline{Sh}_x = 1.53 \frac{Re_x^m}{2x/D + 2h/D + 1.39} Sc^{0.42} \tag{S6}$$

with

$$m = 0.695 - \frac{1}{2x/D + (2h/D)^{1.33} + 3.06}$$

With the bounds of validity:

$$2 \leq x/D \leq 25$$
$$2 \leq h/D \leq 10$$
$$3000 \leq \mathrm{Re} \leq 90{,}000$$

where the factor of two is introduced to account for the hydrodynamic slot width[2]. We also provide the correlation for a gas round jet mass transfer as:

$$\overline{Sh}_x = \frac{1 - 1.1/(x/D)}{x/D + 0.1(h/D - 6)} F(\mathrm{Re})\mathrm{Sc}^{0.4} \tag{S7}$$

with

$$F(\mathrm{Re}) = 2[\mathrm{Re}(1 + 0.005 \cdot \mathrm{Re}^{0.55})]^{0.5}$$

With the bounds of validity:

$$2.5 \leq x/D \leq 7.5$$
$$2.0 \leq h/D \leq 12$$
$$2000 \leq \mathrm{Re} \leq 400{,}000$$

For the dynamic Gas quenching, we use the refitted Chin et al. correlation from [4]

$$Sh_x = a \cdot Sc^{\frac{1}{3}} \cdot Re_x^{\alpha} \cdot \left(\frac{x}{B}\right)^{\gamma} \cdot \left(\frac{h}{B}\right)^{\alpha} \tag{S8}$$

with the following parameters:

- Laminar boundary layer regime ($1 < |x/B| < 4$):

$$\mathrm{a} = (3.15 \pm 0.25) + (-3.00 \pm 0.25)\theta + (0.75 \pm 0.07)\theta^2 \tag{S9}$$
$$\alpha = (1.22 \pm 0.04) + (-0.26 \pm 0.02)\theta$$
$$\beta = (-0.64 \pm 0.04) + (0.12 \pm 0.03)\theta$$
$$\gamma = (-4.43 \pm 0.26) + (3.56 \pm 0.35)\theta + (-0.71 \pm 0.12)\theta^2$$

- Wall jet regime ($8 < |x/B|$):

$$\mathrm{a} = (0.083 \pm 0.029) + (0.038 \pm 0.018)\theta \tag{S10}$$
$$\alpha = (0.84 \pm 0.02) + (-0.061 \pm 0.014)\theta$$
$$\beta = (-0.97 \pm 0.02) + (0.24 \pm 0.01)\theta$$
$$\gamma = (0.69 \pm 0.09) + (-0.88 \pm 0.08)\theta + (0.25 \pm 0.02)\theta^2$$

Because the parameters were refitted it is questionable whether the original bounds of the correlation are still valid. However, the slot width should be around $50\ \mu m$ – $1\ mm$, the height around 0.3 - $3\ mm$ and the air flow velocity from $10-250\ m/s$. These are the values that were used for the refitting.

For liquid round jet in antisolvent quenching, we use the relation for liquid round jets at low Reynolds numbers[5]

$$\overline{Sh_x} = 1.648 \cdot Re_x^{\frac{1}{2}} \cdot Sc^{0.361} \tag{S11}$$

which remains valid for Reynolds numbers below 2000-4000.

For spin coating applied in gas or antisolvent quenching of a round jet with diameter d impinging on a round disk with diameter D rotating at angular velocity of ω, we found the relation[6]

$$\overline{Sh_d} = 0.083 \cdot Re_j^{0.2895} \cdot Re_\omega^{0.3006} \cdot Sc^{0.2663}$$

where $Re_\omega = \omega D^2/\nu$ and $Re_j = du_0/\nu$ are the Reynolds numbers associated with the disk with and the nozzle respectively. With the bounds of validity

$$800 \leq Re_j \leq 5000$$
$$75{,}000 \leq Re_\omega \leq 570{,}000$$
$$65 \leq \mathrm{Sc} \leq 225$$

A comparison of the average mass transfer coefficients and Sherwood correlations included in SupersatRN-C is shown in Figures **S8 -S9**. These are direct, important visualizations of the order of magnitude of the quantities that illustrate how the mass transfer depends on the size of the averaged area. In Figures **S10-S11**, by the use of **Equation S4**, the local representations of the Sherwood correlations and mass transfer correlations are depicted. The most important feature is the locally high mass transport coefficients (and Sherwood numbers) achieved by the narrow slot-jet correlation, which leverages in dynamic gas quenching (black solid line). It also shows the inhomogeneity of the mass transfer coefficient according to this experimentally-tested correlation confined to a region of centimeters (or even millimeters if the slot widths are reduced and higher mass transfer coefficients) are required. Consequently, to achieve a scalable fabrication, the drying process must be fine-tuned such that the crystallization happens always occurs right under nozzle center (that is close to $x = 0$). It is also visible that, even though the laminar mass transport coefficient is lower, it is very spatially homogeneous, which is beneficial for scaling in vacuum quenching. In this quenching technique, high supersaturation is achieved by an exponential decrease in pressure (instead of increasing the air speed).

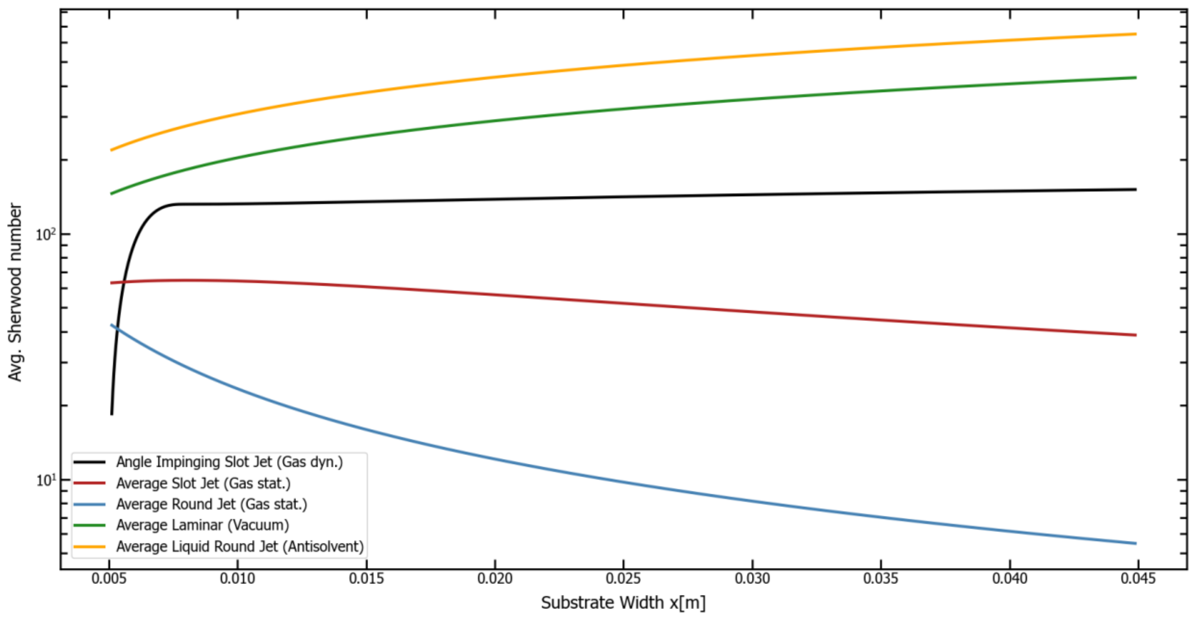

Figure S8 Average Sherwood correlations plotted over the dimension, over which they are averaged. The parameters are $h = 1$ mm, $D = 1$ mm, $u_0 = 100\ m\ s^{-1}$ for the gas quenching (Dry air and DMF gas) and $h = 1$ mm, $D = 1$ mm, $u_0 = 0.1\ m\ s^{-1}$ for antisolvent quenching (CB in DMF).

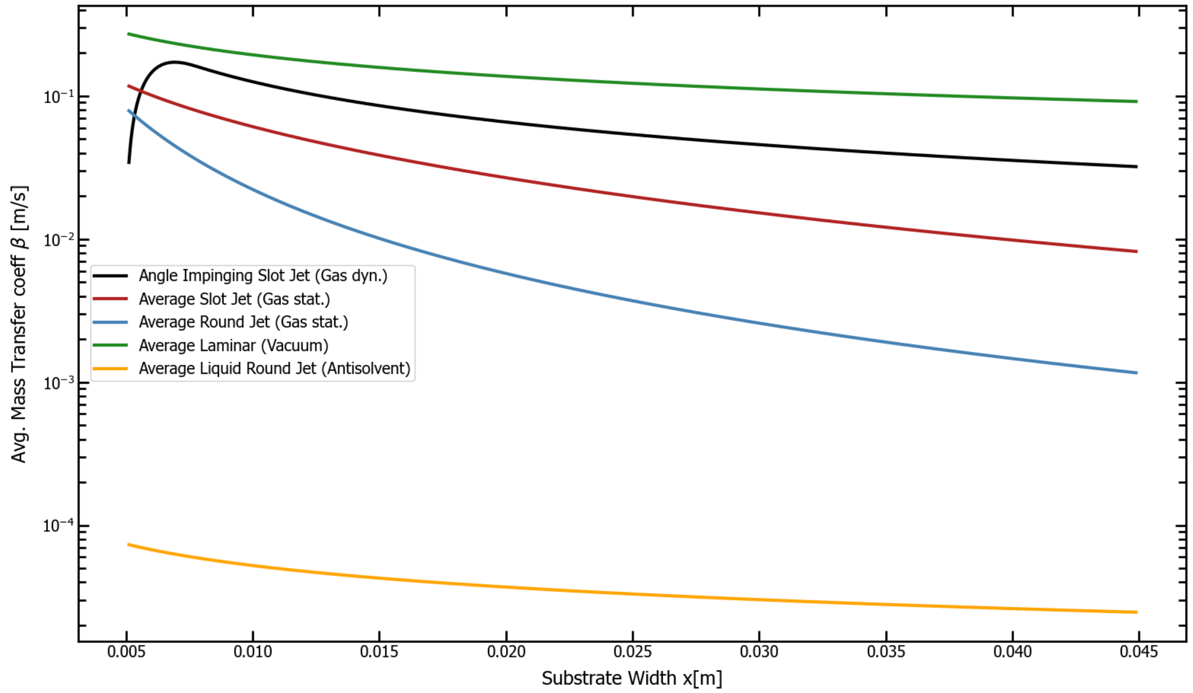

Figure S9 Average Mass transfer coefficients plotted over the dimension, over which they are averaged. The parameters are $h = 1$ mm, $D = 1$ mm, $u_0 = 100\ m\ s^{-1}$ for the gas quenching (Dry air and DMF gas) and $h = 1$ mm, $D = 1$ mm, $u_0 = 0.1\ m\ s^{-1}$ for antisolvent quenching (CB in DMF).

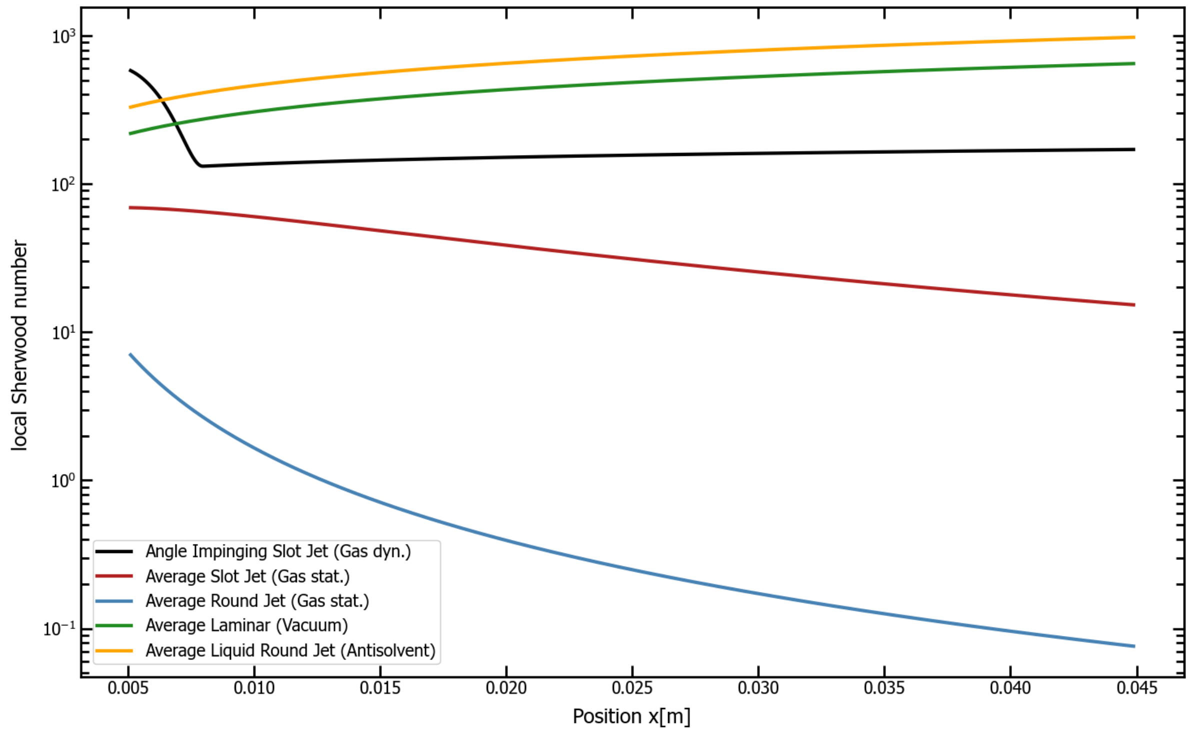

Figure S10 Local Sherwood numbers plotted over the position. The parameters are $h =$ 1 mm, $D =$ 1 mm, $u_0 = 100\ m\ s^{-1}$ for the gas quenching (Dry air and DMF gas) and $h =$ 1 mm, $D =$ 1 mm, $u_0 = 0.1\ m\ s^{-1}$ for antisolvent quenching (CB in DMF).

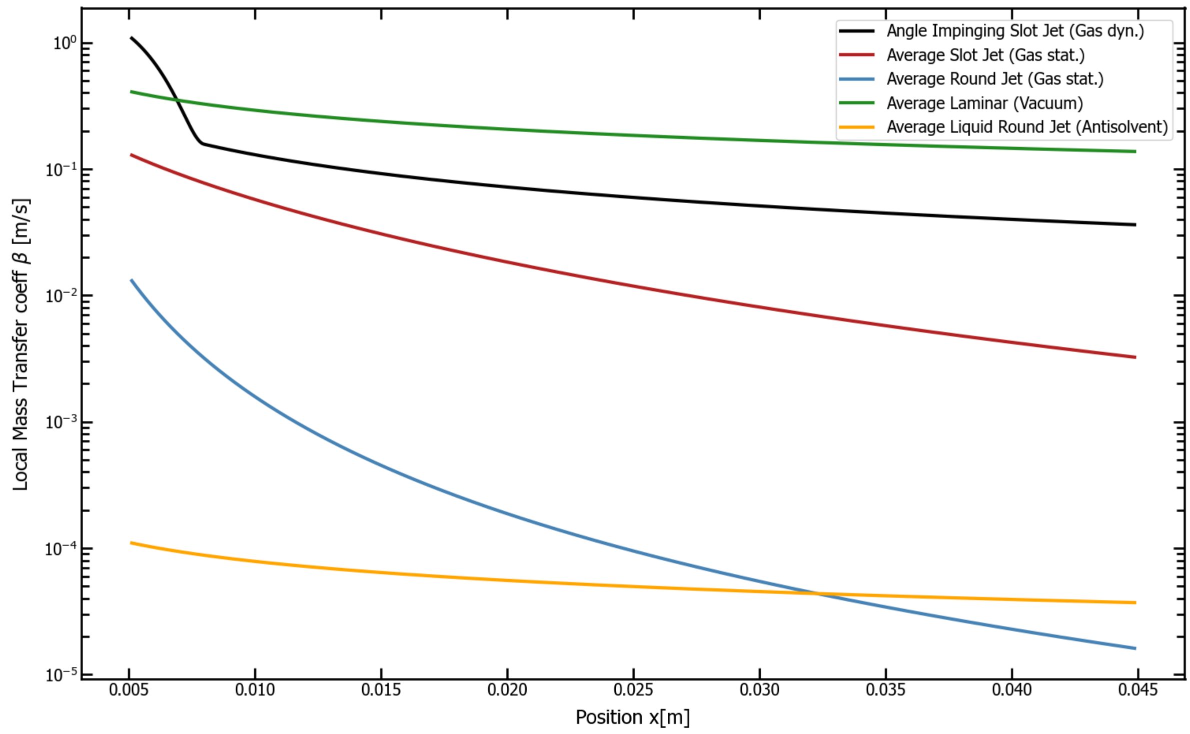

Figure S11 Local Mass transfer coefficients plotted over the dimension, over which they are averaged. The parameters are $h =$ 1 mm, $D =$ 1 mm, $u_0 = 100\ m\ s^{-1}$ for the gas quenching (Dry air and DMF gas) and $h =$ 1 mm, $D =$ 1 mm, $u_0 = 0.1\ m\ s^{-1}$ for antisolvent quenching (CB in DMF).

Supplementary Note 3: Test of isothermal assumption

In order to test the isothermal assumption, we consider a film of DMF with a thickness, $d = 10$ μm, that is dried in the vacuum chamber at a rate similar to the one shown in Figures 3 and S6, which is about $\dot{d} = 1$ μm s^{-1}. We then assume an equilibrium state, where the amount of heat transferred through the film to the film surface is equal to the one used for the evaporation of DMF molecules at the film surface. The temperature difference between the bottom and the top surfaces of the film is then given as

$$\Delta T = \frac{q}{k} d = \frac{\Delta H_{vap}\, J_{DMF}}{k} d = \frac{\Delta H_{vap}\, \tilde{\rho}_{l,DMF}}{k} \dot{d} d,$$

where $k = 0.185\ Wm^{-1}K^{-1}$ is the thermal conductivity of DMF[7], $\Delta H_{vap} = 43$ kJ mol^{-1} is the enthalpy of vaporization[8], $\tilde{\rho}_{l,DMF} = 1.3 \cdot 10^{4}$ mol m^{-3} [8] . With these quantities, we get the estimation $\Delta T = 3 \cdot 10^{-4}$ K and conclude that the isothermal approximation is sufficiently accurate for the drying process inside of the vacuum chamber.

Supplementary Note 4: Interferometric dynamic measurement on a vacuum chamber

We perform an interferometric measurement of perovskite solution film thickness during vacuum quenching. The principal methodology was introduced by an earlier work[1] using a laminar air flow and is therefore not treated in great detail. Here, we apply the same methodology by adding a photodiode and green laser on top of the vacuum chamber penetrating through the transparent top glass (see **Figures S4**a and b). A silicon substrate is blade coated with the investigated solution prior to the start of the vacuum process. The corresponding interference signal is depicted in Figure S5. Figure S6 shows the resulting drying curves that are calculated from the temporal positions of the maxima of the recorded osscillations. We find fundamental differences between the pure solvent film (black dots) and the perovskite solution films. Comparing the MAPI film and the pure solvent film, we find that there is a difference in drying rate at the end of the drying process, which we explained in detail earlier[1]. It is caused by an accerlation of the drying due to a shift of the drying front (pure solvents) and a reduction in solvent concentration, respectively. We further find a further deceleration of the drying in the double cation solutions, which we attribute to the formation of an intermediate phase and an associated reduction in the solvent activity. For determining the activity reduction, we fit the drying curve, first of all, for the precursor containing solely DMF and DMSO and find $\gamma_{DMSO} = 0.17$ (see Figure S7). We then determine the activity of $\gamma_{GBL} = 0.11$ by fitting the data of the precursor containing the full solution (resulting in Figure 3b).

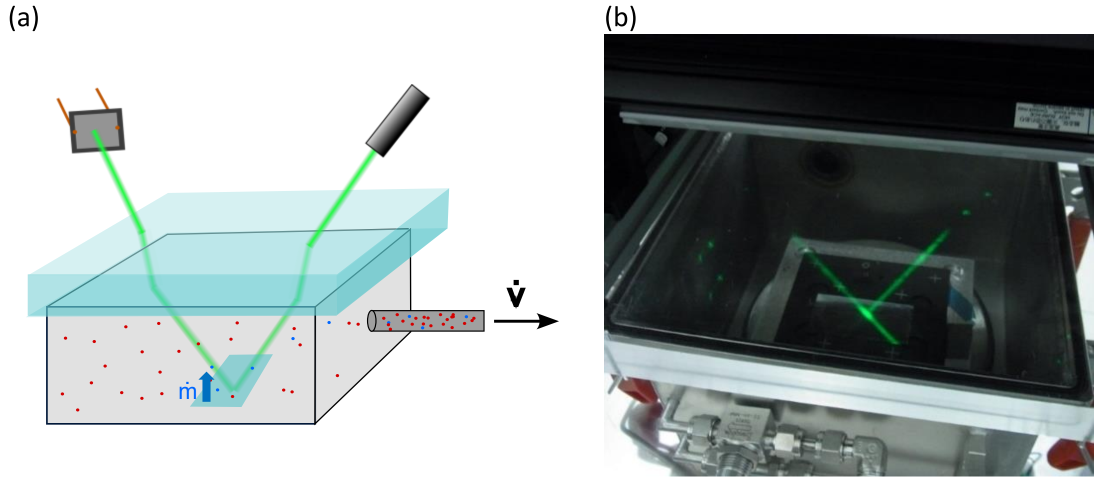

Figure S4 (a) Sketch of vacuum chamber with added possibility to perform interferometric measurements of the dynamic thickness decrease. (b) Photograph of the vacuum chamber during the vacuum quenching. The laser beam is clearly visible because, due to the reducing the pressure, the humidity of the air precipitates as fog.

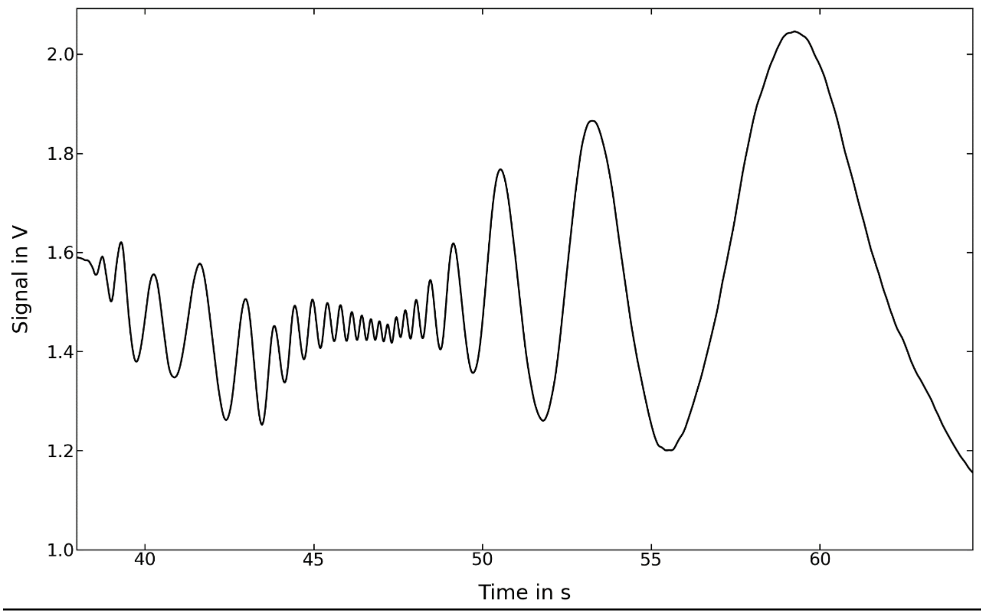

Figure S5 Typical interference signal as measured by the photodiode during vacuum quenching. The temporal positions of the maxima and minima are used to calculate the film thickness at every position in time as introduced in an earlier work [cite].

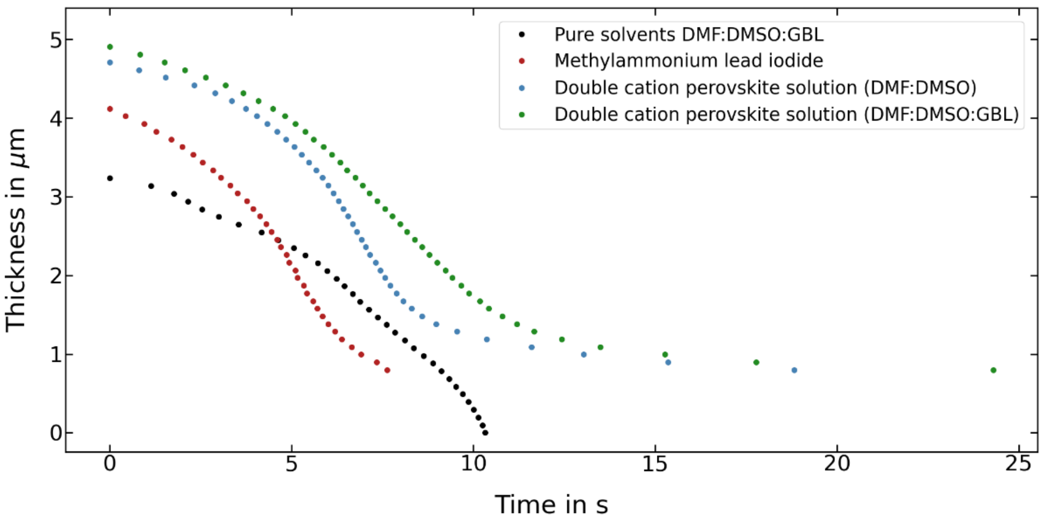

Figure S6 Dynamic thickness evolution of a pure solvent film of DMF:DMSO:GBL (volume ratio 0.39,0.18,0.43), a double cation precursor with the solvents DMF:DMSO (volume ratio 4:1) and DMF:DMSO:GBL (volume ratio 0.39,0.18,0.43).

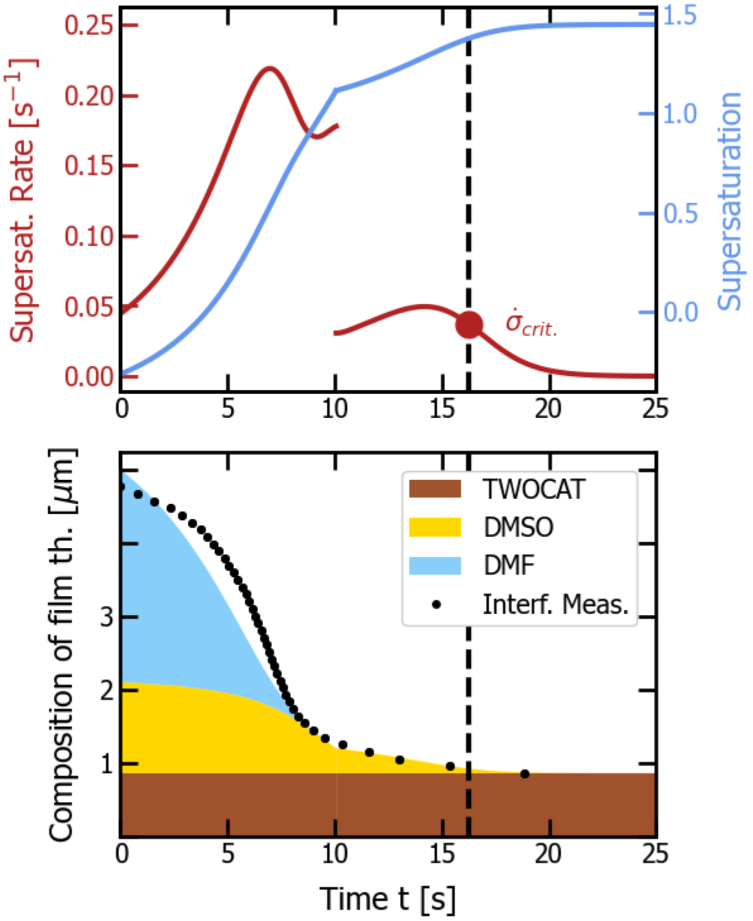

Figure S7 Dynamic thickness evolution of double cation precursor dissolved in pure DMF:DMSO. The activity of DMSO in the second drying regime, starting at 1.5 μm is fitted obtaining $\gamma_{DMSO} = 0.16$.

Supplementary Note 5: Evaporation mechanisms at low pressure

During vacuum quenching, the atmospheric pressure in the chamber is drastically reduced. It is therefore possible that vacuum quenching involves fundamentally different mass transfer dynamics than gas quenching. Herein, we argue that, in our view, these are not decisive for vacuum quenching. We start by discussing, at which pressure the solvent film should start boiling. It is true that the vapor pressure of DMF at room temperature is above 10^3 Pa and of DMSO or GBL above 10^2 Pa. So, just regarding the vapour pressures, we should observe boiling in these solvent film within the vacuum chamber. However, when performing experiments with pure solvent films in the vacuum chamber, we never observed any gas bubbles forming at the liquid-substrate interface, which is one of the clear indications of boiling. On the contrary, we observed that isolated drops of solvent remained on the substrate for minutes after the opening of the valve, which we attribute to the decay of air speed over the substrate reducing the mass transfer close to zero. Considering the high surface activation of the substrate, we conclude that the nucleation energy of solvent gas bubbles, which is necessary for boiling, is never exceeded because maintaining the liquid-solid interface requires less energy. On top of that, if boiling occurred (potentially through the edges of the film), we would expect to see a discrete jump in the drying rate whenever the boiling point of the mixture is reached and, furthermore, much faster drying dynamics than are actually observed. (Mass transfer in nucleation boiling can be described according to well-known correlations[2,3]). When the precursor salts are added to the solvents, a further reduction in boiling point, due to boiling point elevation, is to be expected. We would therefore consider the occurrence of boiling in the perovskite solution even more unlikely.

Let us further investigate the hypothesis different mass transfer dynamics. It is well-known that a fluid can change properties at low pressures, directly impacting the mass transfer [9]. However, we want to demonstrate that we do not leave the viscous air flow regime, where the gas flow is governed by the gas viscosity, at any time during vacuum quenching. This is equivalent to stating that the Knudsen number, Kn, is smaller 0.01. We calculate the smallest Knudsen number as Kn $\approx\ 10^{-7}p_{\mathrm{atm}}/p$ and conclude that we remain in the viscous flow regime if $p >$ 1 Pa.

We continue by investigating the non-equilibrium drying regime, where a Knudsen layer forms over the film surface due to a fundamental kinematic limitation drying[10]. In this (extreme) case, Schrage evaporation limits the evaporation of liquid film due to molecular interaction by [11]

$$\omega_i \approx \sigma \sqrt{\frac{1}{2\pi m_i RT}}\,(p_i - p_k) \qquad \text{(S12)}$$

where $0 < \sigma \leq 1$ is the evaporation coefficient, m_i is the molecular mass of the solvent and p_k is the partial pressure very close to the surface. However, we will show that for vacuum quenching $\omega_i = 0$, due to $p_i = p_k$ at all times. This is equivalent to stating that $\sigma^2 RT \gg\ 2\pi m_i \beta_{ig}^2 (p_{atm}/p)^{2(1-n)}$ meaning that the Schrage evaporation is faster than the solvent transport by diffusion (we assume constant air speed velocity for simplicity). For a typical $\beta_{ig} = 10^{-2}$ m/s, (corresponding to $u_0 \approx 5\ \mathrm{m/s}$) and $\sigma = 1$, this is equivalent to $p \gg 10^{-3}$ Pa.

Supplementary Note 6: Simulation of air flow during vacuum quenching

The used vacuum chamber is depicted in **Figure S12**. Notably, the pressure is not measured inside of the main chamber V_1, but in the external volume V_2. The pressure inside V_2 was fitted with the equation

$$p(t) = (p_{atm} - p_0)\exp(-k_p t) + p_0 - q_p t \tag{S13}$$

(extending Equation 11 of the main manuscript, a linear term was added to account for the final continuous decrease in pressure. This Term is also accounted for in the VacuumDynamics in SuperSatRN-C). For the Volume V_2 we further define the average flow equation

$$\frac{dp}{dt} = \frac{d\tilde{p}_2}{dt} RT = \frac{\dot{n}_{in} - \dot{n}_{out}}{V_2} RT \tag{S14}$$

Where $\dot{n}_{in}$ is the flux of particles into the volume and $\dot{n}_{out}$ is the flux of particles out of the volume. From this we calculate

$$\dot{m}_{out} = \dot{m}_{in} - M_{air} \frac{dp}{dt} \frac{V_2}{RT} \tag{S15}$$

with the numbers $V_2 = 2.7 \cdot 10^{-4}\, m^3$, $R = 8.3 \frac{J}{Kmol}$, $T = 293K$, $M_{air} = 28.9 \cdot 10^{-3}$ kg/mol, and dp_2/dt as the derivative of Equation S13.

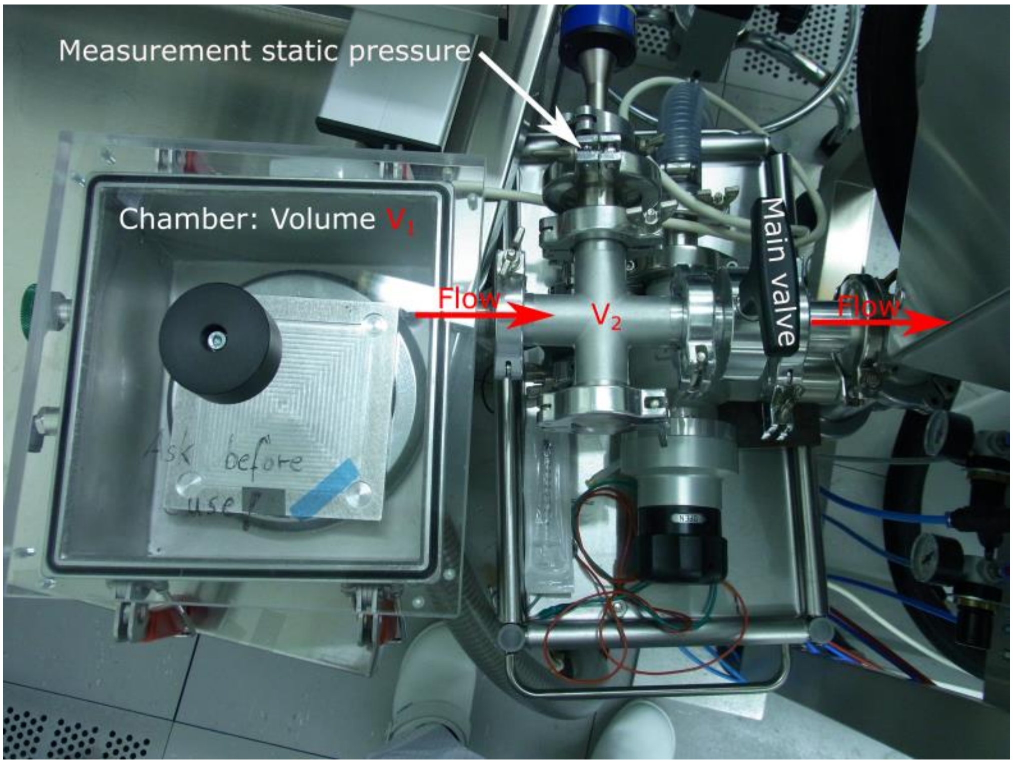

Figure S12 Photograph of used vacuum chamber. The sample is placed in the middle of the main chamber (volume V_1). When the main valve (right side of the picture) is opened lead to about 4m of already pumped down pipe, the pressure decrease is measured in volume V_2) resulting in the data shown in Figure S13.

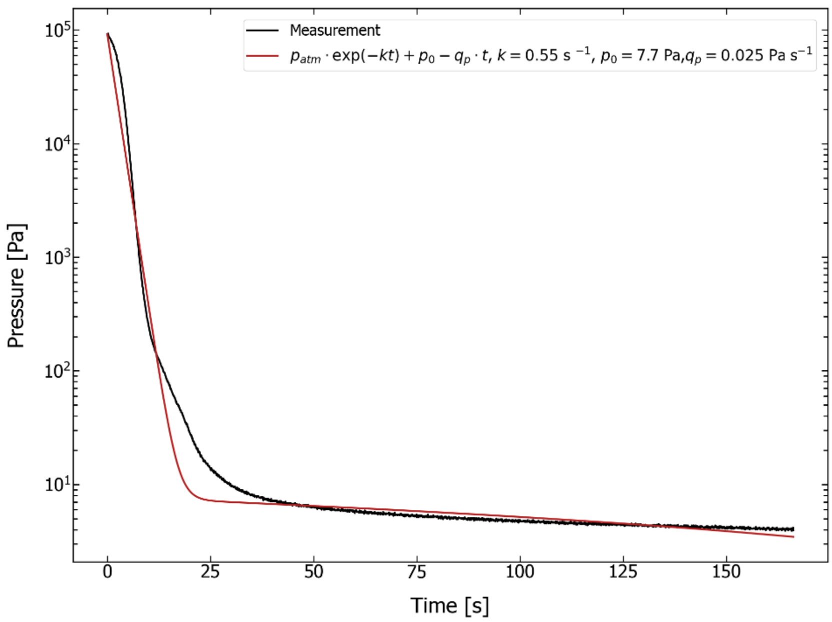

Figure S13 Pressure decay measured for the vacuum process (empty chamber) shown in Figure S12. Initially, the pressure decreases very fast (exponentially) until it reaches a quasi-steady regime at round 5.4 Pa. A linear decreasing term was added to account for the further, very slow, drift in pressure reduction. For the fit a relative error of 10% of the respective pressure value was used.

In order to estimate the evolution air flow velocity during gas quenching over the substrate, we use Ansys® Fluent 2023 R2 in the k-epsilon-Method (energy off). The vacuum chamber is replicated exactly with a CAD drawing, loaded into Ansys Design Modeller and meshed afterwards (Compare **Figures S12** and **S14**). Then, the operating pressure is set to 1 Pa, to model the environment of the vaccum pipe connected to the chamber, an outlet is set to the end of the pipe at which **Equation S15** is set as a boundary condition. The section up to the valve is then patched to 101325 Pa (1 atm) after hybrid inizialization. Suitable graphics and a point of interest for the velocity magnitude over the sample is the selected. The calculation is started with a fxied stepsize of 0.0025 s and 40 steps per iteration. The calculation was carried out for about 60 h real time on 4 cores of an INTEL® Core ™ i7-11800H processor at 2.30 Ghz. **Figures S13-S15** show the simulation at one moment in time (t = 2.3s). The final, calculated air flow velocity measured at one point about 2 cm over the substrate is visible in **Figure S16**, where we have fitted Equation 11 to the simulated data.

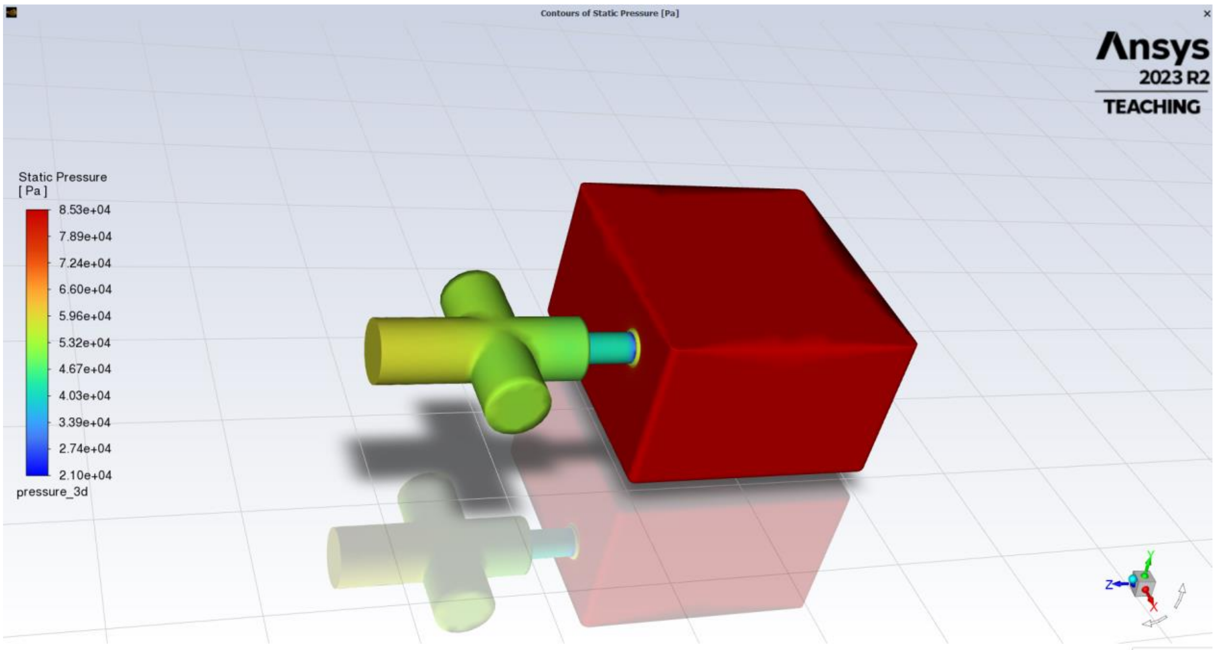

Figure S14 Pressure distribution in vacuum quenching as Simulated by ANSYS Fluent on the modelled vacuum chamber. The physical time is t = 2.3s

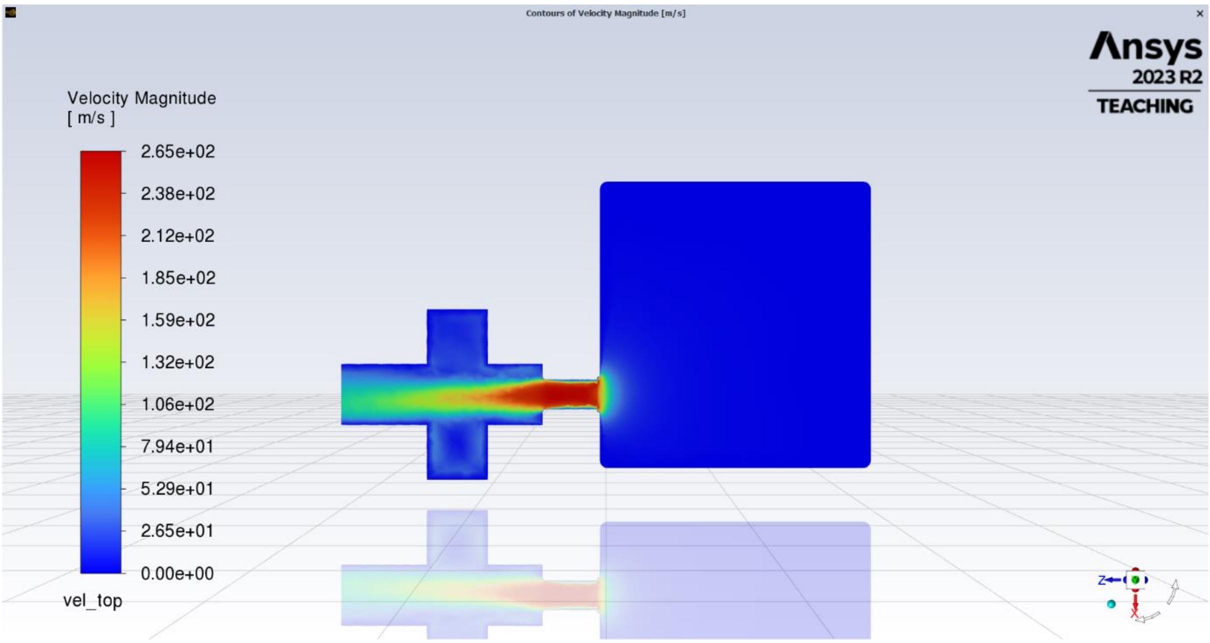

Figure S15 Velocity distribution in the chamber seen from top. The highest velocity (265 m/s) is reached in the narrow connection between the chamber and the vacuum tube. The slight asymmetry is attributed to the asymmetry of the opening of the vacuum chamber. The physical time is t = 2.3s.

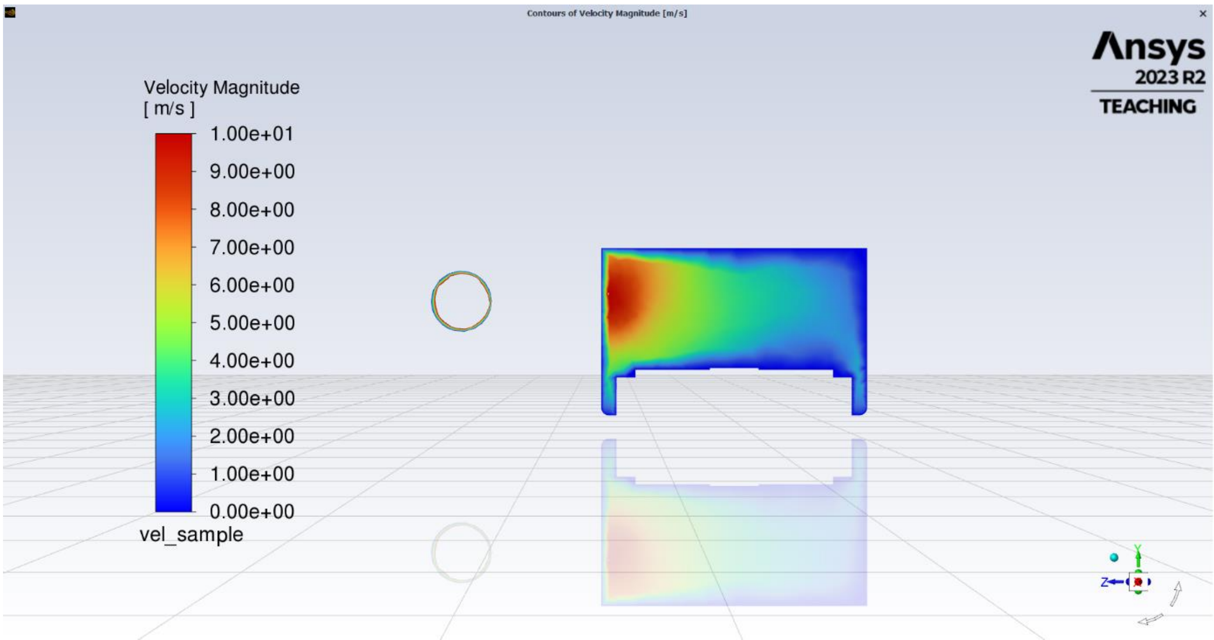

Figure S16 Velocity distribution on a cross section of the chamber shown in **Figure S13** The air is sucked out on an opening on the left-hand side. The sample is visible on the elevated table in the middle. In a point, over the sample, the velocity magnitude is recorded, yielding **Figure S16**. The physical time is t = 2.3s.

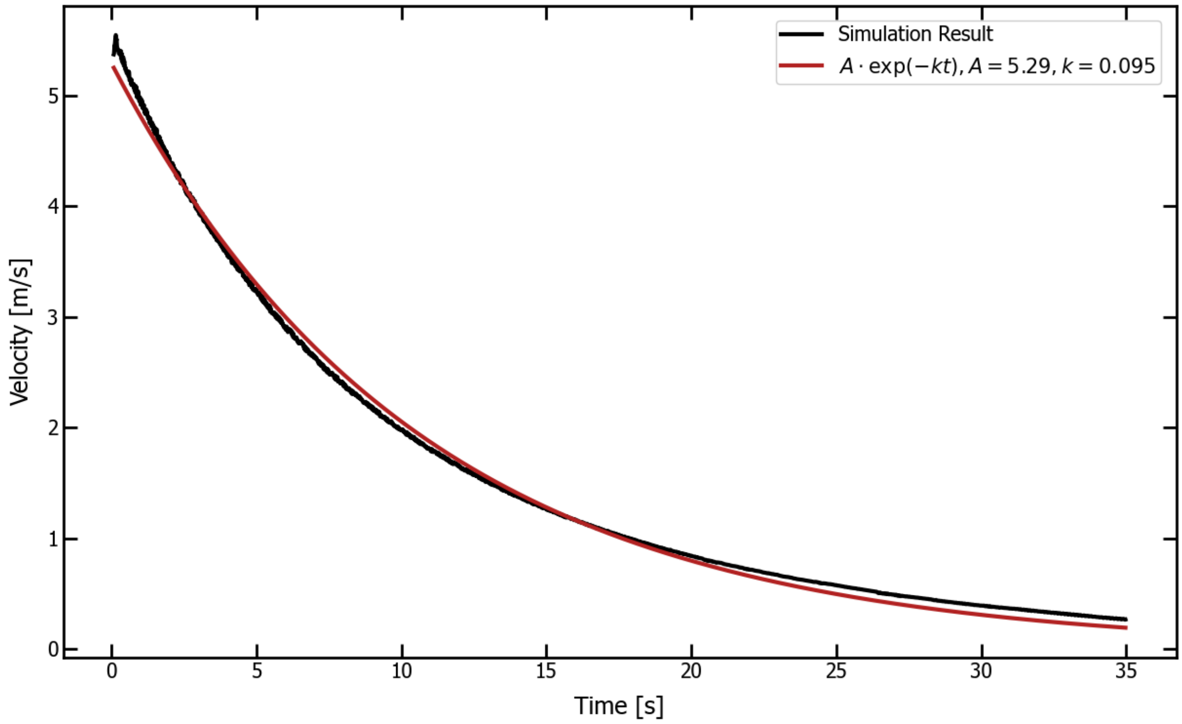

Figure S17 Velocity magnitude (measured 2 cm over the sample) over time in vacuum quenching as simulated by ANSYS Fluent on the modelled vacuum chamber (black solid line) and a fit of Equation (red solid line). A short time (<0.1s) is needed for stabilization of the air flow, which is neglected in the fit and not shown here.

Supplementary Note 7: Influence of certain parameters on the critical supersaturation

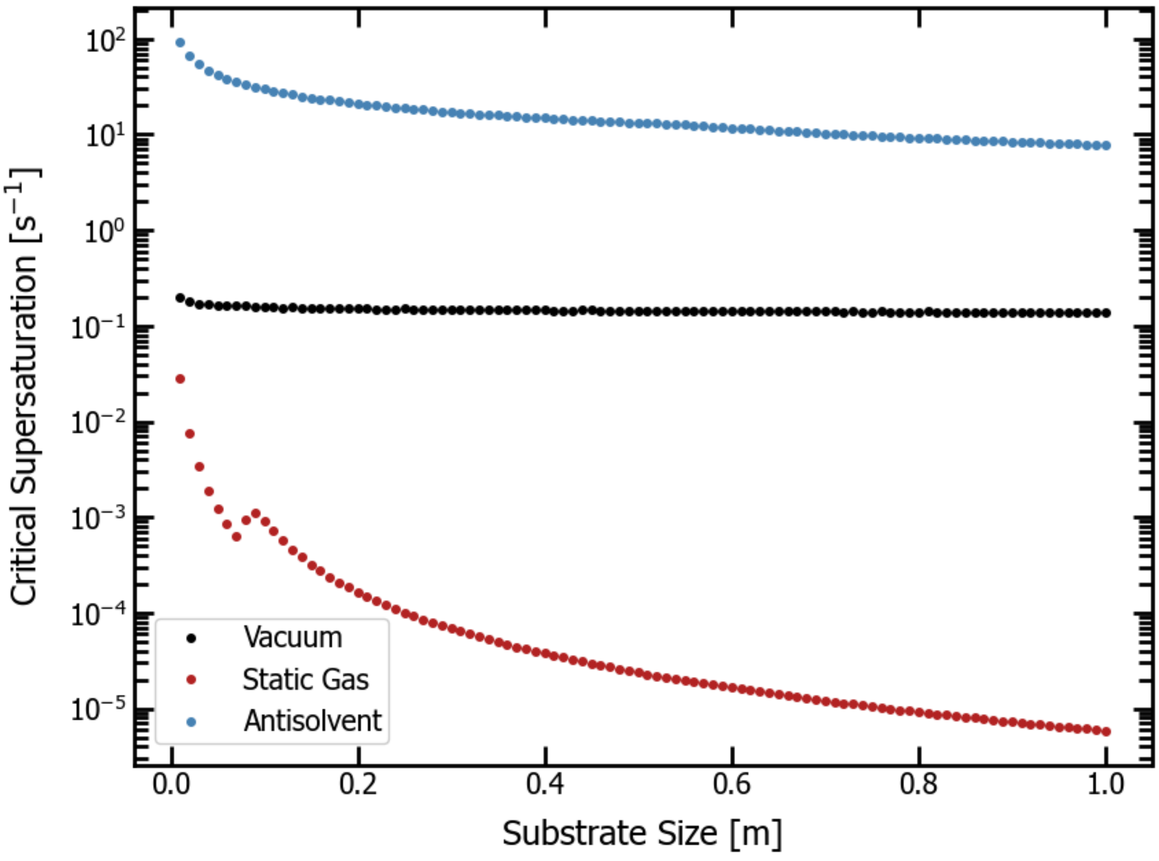

Figure S18 Scaling behavior of different quenching methods: Supersaturation rates as predicted by SupersatRN-C are plotted over varying substrate size. It is apparent that antisolvent quenching and static gas quenching lead to a larger scaling loss, which is mainly due to the decay of Sherwood numbers, which is balanced by the exponential acceleration of drying rate in vacuum quenching. It however has to be noted that a possible saturation of the vacuum chamber with solvent gas is not yet included in the model, so these data only apply to the case where all solvent gas is extracted fast enough.

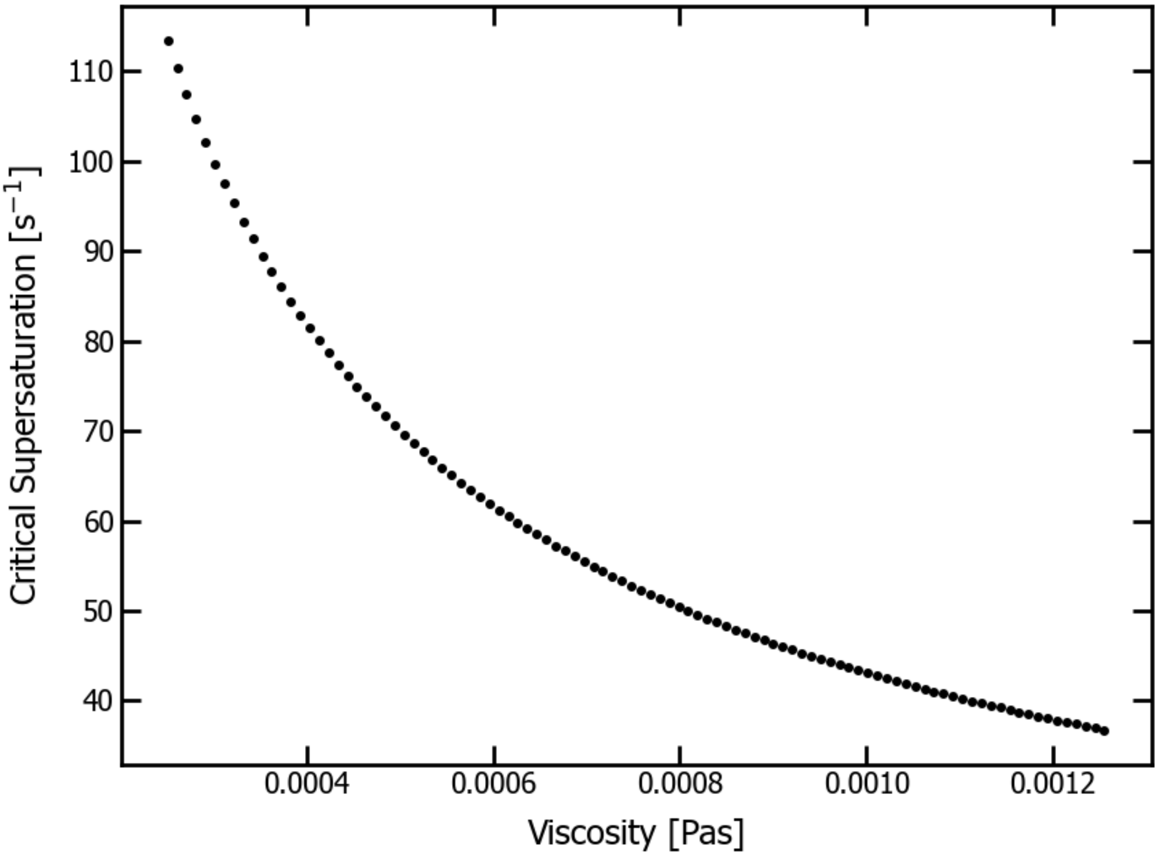

Figure S19 Influence of solvent viscosity on the reached supersaturation rate in MAPI quenching. It is assumed that q_c is the same as for MAPI and CB. So, these data represent a hypothetical solvent with the same properties of CB, however a variation in viscosity (The true viscosity is about 0.8 mPas).

Supplementary Note 8: Measurement of perovskite thicknesses.

The final thickness of the crystallized perovskite thin films was measured for both precursor systems on multiple substrates by Dektak profilometry. The results are shown in Figure S18.

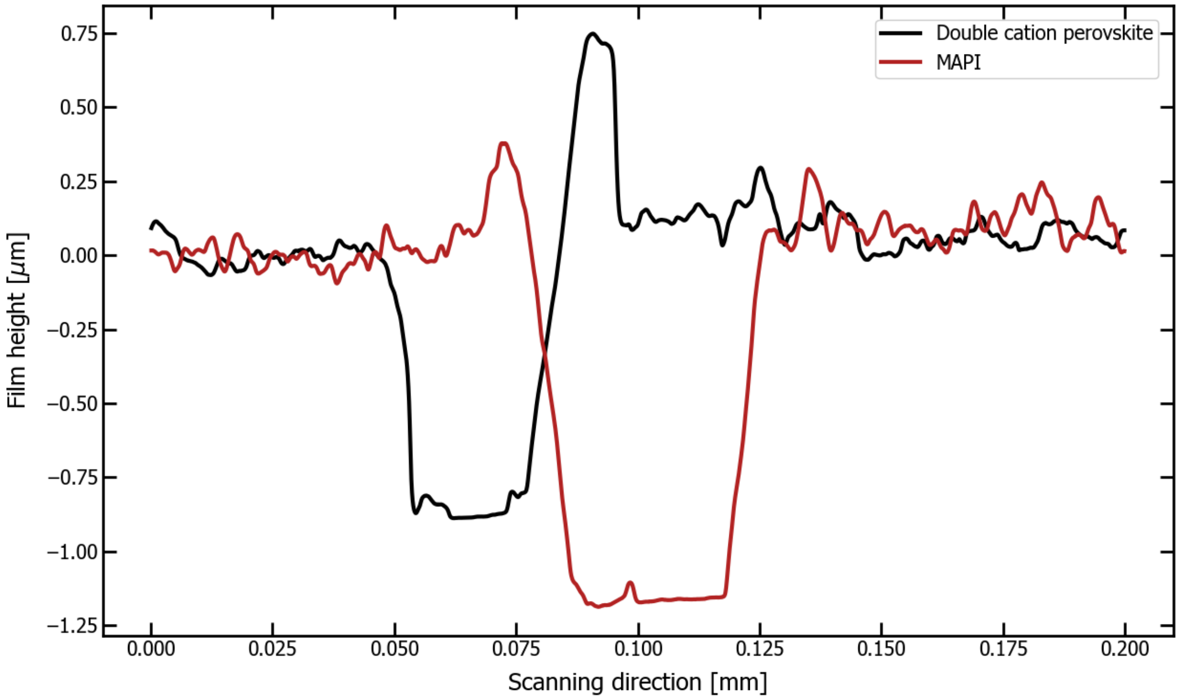

Figure S20 Dektak thickness measurements of MAPI and double cation films used for the interferometric measurements. The dip is caused by a scratch in the thin film. The data was averaged on the film surface and set to zero. The respective film thicknesses are $1.18\mu m$ and $0.86\ \mu m$.

Supplementary Note 9: Generalization of Software

Some of the equations shown in the main manuscript were generalized in the software package to add the capability to combine multiple antisolvents and/or perovskite systems in one solution. Essentially, the calculated quantities are generalized by performing an average in accordance with the volume ratios of solutes $r_j = V_j/V_{\text{tot, solutes}}$ and $r_i = V_i/V_{\text{tot,solvents}}$. This then culminates in the generalization of **Equation 5**

$$C_0 = \sum_{s,Solutes} \sum_{i,Solvents} r_s r_i \left(\bar{C}_{0,s,i} - \sum_{j,antisolvents} q_{r,s,i,j} \frac{N_j}{\sum_{k,Solvents} s_{jk} N_k} \right) \tag{S16}$$

In the SupersatRN-C program, we calculate the supersaturation rate numerically. In this way, possible sources of error can be eliminated.

Bibliography for Supplementary

[1] S. Ternes, T. Börnhorst, J. A. Schwenzer, I. M. Hossain, T. Abzieher, W. Mehlmann, U. Lemmer, P. Scharfer, W. Schabel, B. S. Richards, U. W. Paetzold, *Adv Energy Mater* **2019**, *9*, 1901581.

[2] VDI e. V., *VDI-Wärmeatlas - 2013*, Springer Vieweg, Berlin, Heidelberg, **2013**.

[3] T. L. Bergman, F. P. Incropera, *Fundamentals of heat and mass transfer.*, Wiley, **2011**.

[4] S. Ternes, J. Mohacsi, N. Lüdtke, H. M. Pham, M. Arslan, P. Scharfer, W. Schabel, B. S. Richards, U. W. Paetzold, *ACS Appl Mater Interfaces* **2022**, *14, 9,* 11300.

[5] John H. Lienhard V, *18th National & 7th ISHMT-ASME Heat and Mass Transfer Conference* **2006**, 1.

[6] L. Jiang, Y. Lyu, P. Zhu, W. Gao, Z. Liu, *International Journal of Thermal Sciences* **2021**, *170*, 107097.

[7] M. R. Rodríguez-Laguna, A. Castro-Alvarez, M. Sledzinska, J. Maire, F. Costanzo, B. Ensing, M. Pruneda, P. Ordejón, C. M. Sotomayor Torres, P. Gómez-Romero, E. Chávez-Ángel, *Nanoscale* **2018**, *10*, 15402.

[8] K. Panneerselvam, M. P. Antony, T. G. Srinivasan, P. R. Vasudeva Rao, *Thermochim Acta* **2009**, *495*, 1.

[9] Helmut. Bannwarth, Christine. Ahner, **2005**, 492.

[10] T. Ytrehus, S. Østmo, *International Journal of Multiphase Flow* **1996**, *22*, 133.

[11] R. W. Schrage, *A Theoretical Study of Interphase Mass Transfer* **1953**.
